# Trophic niche overlap between round sardinella (*Sardinella aurita*) and sympatric pelagic fish species in the Western Mediterranean

**DOI:** 10.1002/ece3.8293

**Published:** 2021-11-10

**Authors:** Eneko Bachiller, Joan Giménez, Marta Albo‐Puigserver, Maria Grazia Pennino, Neus Marí‐Mena, Antonio Esteban, Elena Lloret‐Lloret, José María Bellido, Marta Coll

**Affiliations:** ^1^ Marine Renewable Resources Department Institute of Marine Science (ICM‐CSIC) Barcelona Spain; ^2^ MaREI Centre Environmental Research Institute University College Cork Cork Ireland; ^3^ School of Biological, Earth, and Environmental Sciences University College Cork Cork Ireland; ^4^ Centro de Ciências do Mar Universidade do Algarve (CCMAR‐UAlg) Faro Portugal; ^5^ Centro Oceanográfico de Vigo Instituto Español de Oceanografía Vigo Spain; ^6^ AllGenetics & Biology SL. Oleiros A Coruña Spain; ^7^ Centro Oceanográfico de Murcia Instituto Español de Oceanografía San Pedro del Pinatar Spain

**Keywords:** diet dissimilarity, multi‐proxy diet analysis, prey preference, small pelagic fish, trophic interactions, trophic niche width

## Abstract

The northward expansion of round sardinella (*Sardinella aurita*) in the Mediterranean Sea, together with declines and fluctuations in biomass and landings of European sardine (*Sardina pilchardus*) and anchovy (*Engraulis encrasicolus*) observed in recent decades, may suggest potential inter‐specific competition in the pelagic domain. The coexistence of sympatric zooplanktivorous fish species might therefore be exposed in part to trophic niche overlap and competition for food. Combining visual diet characterization under the microscope with DNA metabarcoding from stomach contents of fish collected in spring results show that predation on relatively large krill is equally important for sardinella than for the other two niche overlapping species. Furthermore, an important overlap is found in their isotopic niche, especially with anchovy, using nitrogen (δ^15^N) and carbon (δ^13^C) stable isotopes in muscle tissue. In fact, the three fish species are able to feed effectively in the whole prey size spectrum available during the sampled season, from the smallest diatoms and copepods to the larger prey (i.e., decapods and euphausiids), including fish larvae. Moreover, effective predation upon other large prey like siphonophores, which is observed only when multi‐proxy analyses in stomach contents are applied, might also be relevant in the diet of sardinella. The overlapping diet composition in spring, together with the effective use of food resource by sardinella, can be of special interest in potential future scenarios with warmer water temperature leading to lower zooplankton and/or higher jellyfish availability, where sardinella may take advantage over other species due to its feeding plasticity.

## Graphical abstract

The present study studies the trophic ecology of sardinella, anchovy, and sardine in the NW Mediterranean Sea, combining microscope visual analysis with DNA metabarcoding in gut contents as well as stable isotope analysis in muscle. Results with high taxonomic resolution determined an important degree of trophic niche overlap between the three species, which adapt their feeding to a wide range of available plankton, from the phytoplankton as well as the smallest copepods to larger krill or jelly organisms. Therefore, in the Mediterranean pelagic fish community, the diet plasticity of the recently incorporated (and expanding) sardinella might increase the inter‐specific competition if the food availability changes in the future.

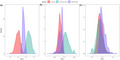

## 1 INTRODUCTION

With the global increase in sea surface temperature (Bopp et al., 2013; Pisano et al., 2020; Séférian et al., 2020), species that prefer warmer waters will increase its northward expansion, as it has been seen for round sardinella (*Sardinella aurita*), not only in the Mediterranean Sea (Sabatés et al., 2006) but also in the eastern Atlantic waters (Zeeberg et al., 2008). How these shifts in their distribution might affect inter‐specific interactions will depend on many factors, but among them, the trophic niche overlap of sympatric species and its trophic interactions are important in many marine ecosystems (e.g., Checkley et al., 2009; Kadin et al., 2019), including the Mediterranean Sea (e.g., Albo‐Puigserver et al., 2016, 2019; Bachiller et al., 2020; Coll et al., 2019).

In this context, the case of the round sardinella (hereafter referred to as “sardinella”) with European sardine (*Sardina pilchardus*) and anchovy (*Engraulis encrasicolus*) has received special attention, mainly since sardine biomass declined and anchovy fluctuated (Pennino, Coll, et al., 2020), while sardinella distribution expanded from the south to northern areas showing higher potential for niche overlap with the other two species in the northern Mediterranean Sea (Francour et al., 1994; Maynou et al., 2020; Sabatés et al., 2006; Sinovcic et al., 2004; Tsikliras, 2008). Since anchovy and sardine are highly commercial fish species in the Mediterranean Sea (FAO, 2018), while sardinella has lower commercial value, a negative result from this overlap could affect fishing activities with socioeconomic effects.

Regarding the feeding ecology, the three small pelagic species are described as planktivorous feeders, mainly preying on copepods, cladocerans, and diatoms, with some seasonal variation (Albo‐Puigserver et al., 2016; Costalago & Palomera, 2014). All three species can switch from particulate to filter feeding (Bachiller et al., 2020; Costalago et al., 2015; Karachle & Stergiou, 2013; Nikolioudakis et al., 2014; Tsikliras et al., 2005), but the wider prey size spectrum observed in sardinella (including diatoms and ingesting also larger prey sizes, such as salps; Albo‐Puigserver et al., 2019) might indicate that it is a more generalist species relative to sardine and anchovy (Bachiller et al., 2020). Besides, when large prey availability is high, an effective opportunistic predation on krill has been observed for sardine and especially in anchovy in the southern part of the Western Mediterranean (Bachiller et al., 2020). However, to understand the potential ecological effects of the expansion of round sardinella in the Mediterranean pelagic ecosystem, we need to assess the niche overlap studying the diet composition in detail for the three species together, which is not common (Albo‐Puigserver et al., 2019; Karachle & Stergiou, 2013).

In the present study, we investigate the feeding ecology of sardinella, and how it relates to two sympatric species, sardine and anchovy, that were described in Bachiller et al. (2020), in the Gulf of Alicante (Geographical Sub‐Area 06—hereafter GSA; FAO, 2018). We combine stomach content analysis under the microscope with DNA metabarcoding, applying a novel diet characterization procedure which deals with the prey quantification limitation of genetic approaches (Amundsen & Sánchez‐Hernández, 2019), and we include stable isotope analyses of carbon and nitrogen (δ^13^C and δ^15^N) that integrate trophic information in a longer timeframe (Nielsen et al., 2018). This way, we determine whether sardinella is mainly a filter feeder, or also an effective particulate feeder, which uses the whole prey size spectrum available. This would mean that this species can have a high degree of diet overlap with the other two sympatric species in the Mediterranean Sea, anchovy and sardine, and might result in potential competition for food, especially in future scenarios with poorer feeding conditions, which could impact the fishing sector and have important socioecological effects. In addition, since we previously documented large proportions of microplastics and parasites in the diet of anchovy and sardine (Pennino, Bachiller, et al., 2020), we also analyzed them in stomach contents of sardinella, which could provide an indirect indicator about its health.

## 2 MATERIALS AND METHODS

### 2.1 Sample collection

**FIGURE 1:**
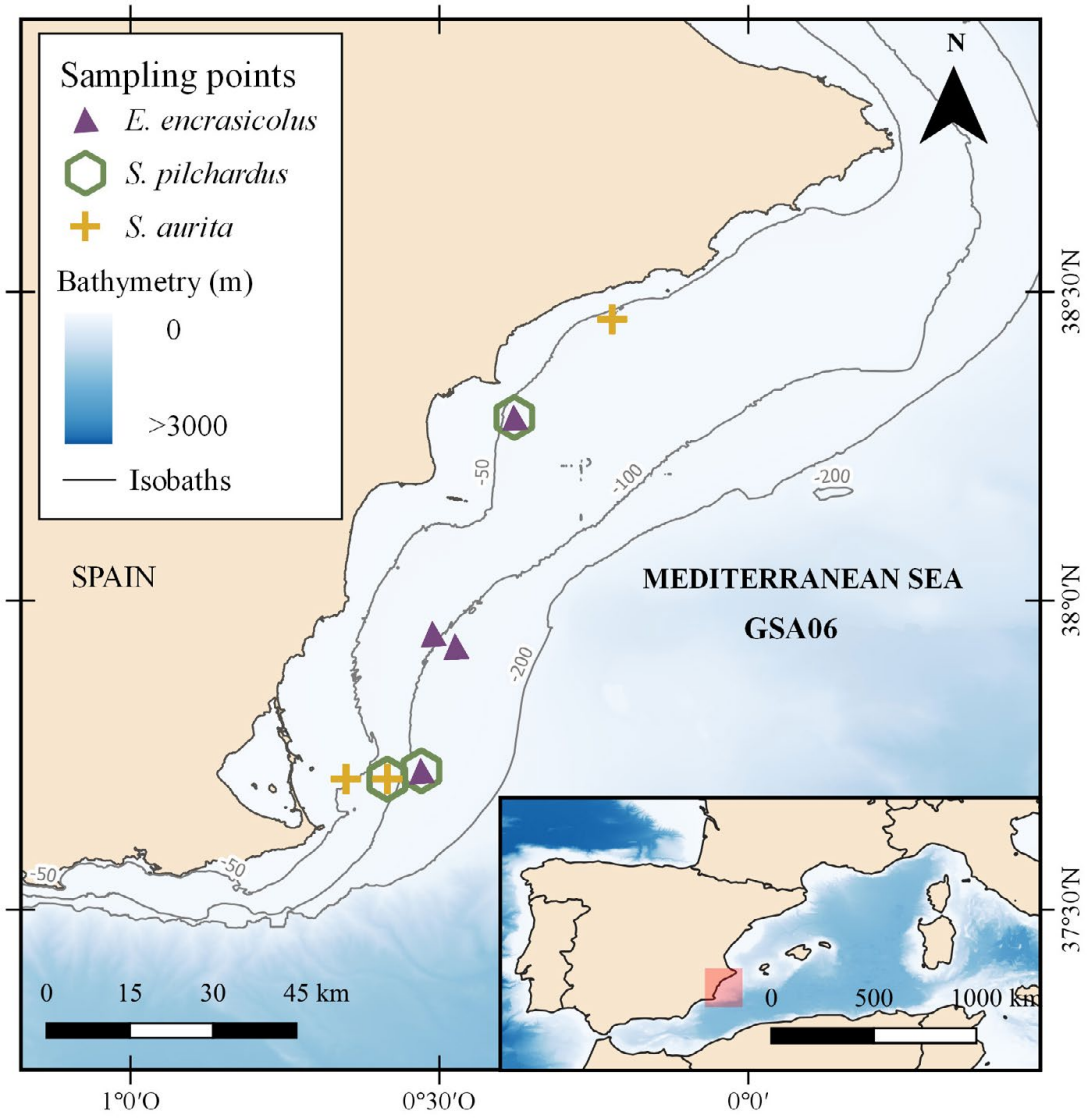
Sampling area in the Western Mediterranean Sea where sardinella (*Sardinella aurita*), anchovy (*Engraulis encrasicolus*), and sardine (*Sardina pilchardus*) were collected. All samples in the marked stations were used for stomach content characterization (under the microscope and with DNA metabarcoding) and for stable isotope analysis (see Table 1 for details). Figure generated with QGIS v. 3.2.1‐Bonn117 (https://qgis.org/en/site/)

Adult fish samples of round sardinella *Sardinella aurita*, anchovy *Engraulis encrasicolus*, and sardine *Sardina pilchardus* were collected in the Western Mediterranean area, in the Gulf of Alicante (GSA 06; FAO, 2018), during the MEDITS (Mediterranean International bottom Trawl Survey) survey (Bertrand et al., 2002; Figure 1, Table1). The MEDITS survey is performed every year using a stratified sampling design based on the coverage of five bathymetric strata (10–50, 51–100, 101–200, 201–500, and 501–700 m). In 2018, we sampled in stations randomly placed within each stratum at the beginning of the monitoring program in mid‐1990s, using a bottom‐trawl GO73 with 20‐mm cod‐end mesh size net (Bertrand et al., 2002). The average vertical opening of the gear was 2 m and its wing span was 18 m. We performed all the tows during daylight hours. We collected the samples of the study during the months of May and June, 2018. Immediately after collection, we measured the total length of fish (TL, in cm) and we extracted the stomachs to be preserved in 96% ethanol for later examination.

**TABLE 1:** Summary of the collected fish samples for sardinella (*Sardinella aurita*), anchovy (*Engraulis encrasicolus*), and sardine (*Sardina pilchardus*), including the total length of fish (TL) and method type applied for the analyses (i.e., the same fish were used for the three methods)

Species	TL_min_–TL_max_ (cm)	Stomach content characterization (*N*)	DNA metabarcoding (*N*)	Stable isotope analysis (*N*)
*S. aurita*	18.70–27.20	31	31	17
*E. encrasicolus*	13.10–16.20	22	22	16
*S. pilchardus*	15.40–17.80	15	13	15

Note

*N* denotes number of samples.

### 2.2 Stomach content characterization under the microscope

Stomach contents of 68 adult (i.e., TL = 13–27 cm) fish samples (Table 1) were analyzed individually, under a NIKON SMZ1270 stereomicroscope with 20–80× amplification. In order to avoid air contamination (e.g., fibers that might then bias the anthropogenic fiber ingestion estimates) during sample processing (Lusher et al., 2017; Nadal et al., 2016; Woodall et al., 2014), microscope analysis was conducted in a “clean room” and with an air extractor placed 20–30 cm above the petri plate containing stomach samples. To avoid contamination between samples, glassware, bench, microscope slide, and dissection equipment (i.e., stainless‐steel scissors, scalpel, and lancet) were rinsed with 96% ethanol prior to each stomach content analysis (Cole et al., 2014).

Only material contained in the stomachs was considered, whereas the contents of the intestine and esophagus were discarded to reduce bias caused by different rates of digestion and cod‐end feeding (Hyslop, 1980). During processing, stomach contents were carefully taken apart and all identifiable prey counted and specified to the lowest possible taxonomic group, not including broken parts of appendixes when quantifying, and categorized into 44 groups (Table S2). Stomach content analyses included characterization of anthropogenic fiber ingestion, defining <2 mm and >2 mm fibers as micro‐ and mesofibers, respectively (i.e., micro‐ and/or mesoplastics); other pollution like paint and/or other plastic remains were all <2 mm and were therefore defined as “other microplastic.” Parasitic organisms found in stomachs were also reported individually in the diet analysis. After microscope analysis, stomach contents were preserved on 96% ethanol for later DNA metabarcoding analysis.

### 2.3 DNA metabarcoding in stomach contents

DNA was extracted from all the stomach content samples of fish analyzed previously under the microscope (Table 1), using the NZY Tissue gDNA Isolation kit as per manufacturers protocol (NZYTech, Lisbon, Portugal). Prior to DNA extraction, vials were shaken by hand to homogenize the stomach contents of each individual sampled fish. An extraction blank was included in every DNA extraction round and treated as a regular sample to check for cross‐contamination.

#### 2.3.1 Zooplankton and diatom characterization in diet

In order to characterize the stomach contents composition in zooplankton and diatom species, samples were analyzed in duplicate, using two different primer sets targeting a fragment of the cytochrome oxidase subunit I (COI) region (Wangensteen et al., 2018) and a fragment of the rbcL chloroplast gene (Vasselon et al., 2018), respectively. In addition, specific blocking primers were designed based on COI sequences to block the amplification of DNA from sardinella, anchovy, and sardine. Library preparation followed the same protocol as described in Bachiller et al. (2020).

Illumina's paired‐end reads were merged with FLASH2 (Magoc & Salzberg, 2011) with a minimum overlap of 30 bp on the expected overlapping region. The CUTADAPT v.1.3 software (Martin, 2011) was used to remove primers and to trim sequences to the amplicon length in both gene regions. Then, sequences from all the samples were quality filtered and pooled using Qiime v.1.9.1 (Caporaso et al., 2010). The VSEARCH software (Rognes et al., 2016) was used to dereplicate the dataset, to cluster sequences using SWARM 2.0 algorithm (Mahé et al., 2015) with a *d* value of 13, and to remove chimeras using the UCHIME algorithm (Edgar et al., 2011). The resulting COI gene sequences were assigned to a custom taxonomic reference database, constructed from the BOLD Public Data Portal (Ratnasingham & Hebert, 2007), using the UCLUST algorithm (Edgar, 2010) implemented in Qiime, with a similarity threshold of the 95%. The rbcL gene sequences were assigned to the R‐Sys reference database (Vasselon et al., 2017) using the naive Bayesian method implemented in RDP (Wang et al., 2007), with a confidence threshold of the 80%. Finally, different filters were applied to the results, taking into account sequence counts and the taxonomic information for zooplankton and diatoms (see Bachiller et al., 2020, for detailed information).

DNA metabarcoding data were obtained from the analyses carried out by All Genetics & Biology S.L. (www.allgenetics.eu).

### 2.4 Stable isotope analyses

Isotopic analyses were performed at the Laboratory of Stable Isotopes of University of A Coruña, Galicia, Spain (Servicio de Análisis Instrumental (SAI)), through an elemental analyzer (Carlo Erba CHNSO 1108) coupled to an isotopic ratio mass spectrometer (Finnigan Matt Delta Plus).

Stable isotope analyses of carbon (δ^13^C) and nitrogen (δ^15^N) were performed in muscle tissue. Fish muscle samples were obtained from 48 individuals (17 sardinella, 16 anchovy, and 15 sardine; Table 1). A small portion of the dorsal muscle without skin from each fish sample was oven dried at 60ºC for 72 h and then pulverized. Muscle powder, 0.80–0.85 mg per fish, was packed into tin capsules.

The isotopic values are reported using delta (δ) notation in parts per thousand (‰), according to the following equation:

(1) $$\delta^{13}C\;\text{or}\;\delta^{15}N=\left[\frac{R_{\text{sample}}}{R_{\text{standard}}}‐1\right]\times 1000$$

where *R* is the ratio of heavy‐to‐light isotope of the sample (*R*_sample_) and the standard (*R*_standard_), respectively, referenced to Vienna Pee Dee Belemnite for δ^13^C and atmospheric nitrogen N_2_ (air) for δ^15^N (Coplen, 2011). USGS40 and l‐alanine from the International Atomic Energy Agency were used, as well as internal acetanilide standards. An accuracy (± SE) of <0.1 and <0.3% was obtained for the standards replicates and samples for the two isotopes, respectively. A C:N ratio greater than 3.5 indicates that lipids are present in the sample, and therefore a correction was applied to the values of δ^13^C according to Post et al. (2007).

The inter‐specific comparability of round sardinella samples, collected in the same week and area, was checked in a preliminary test that showed no change in δ^15^N and δ^13^C between sample locations (*t*‐tests *p*‐values > .1; Table S1).

Differences in δ^13^C and δ^15^N values depending on the total length of sardinella, sardine, and anchovy were tested using lineal regressions. Isotopic niche space and overlap among species were performed using Standard Isotopic Ellipses in the SIBER R‐package (Jackson et al., 2011). Standard isotopic ellipses represent the core isotopic niche for a species (*ca*. 40% of the data). Standard isotopic ellipses areas were corrected for small sample sizes (SEA_C_) to be able to compare between species. Their Bayesian equivalent (SEA_B_) was also computed to have a measure of uncertainty through computing credible intervals around the measurement (Jackson et al., 2011).

### 2.5 Diet analyses

#### 2.5.1 Prey composition

Microscope analysis (i.e., detailed prey characterization) and DNA metabarcoding (i.e., presence–absence information of zooplankton and diatoms OTUs) were combined in order to determine the diet composition. Accordingly, for diet analyses, taxonomic groups were classified into three categories: (1) groups determined under the microscope and detected with DNA metabarcoding; (2) groups determined under the microscope but not detected with DNA metabarcoding; and (3) groups detected with DNA metabarcoding and not determined under the microscope.

The diet composition was first explored using numerical and weight percentages of prey groups relative to total prey consumption based on prey groups determined under the microscope (i.e., categories 1 and 2). For abundances of partially undetermined taxonomic levels, such as “Unidentified Calanoids,” “Unidentified Decapods,” and “Unidentified Euphausiids,” the present study proposes a new correction method, breaking down each group into the detected corresponding species by DNA metabarcoding (hereafter referred to as “corrected diet characterization”). These “uncertain” species taxa were re‐assigned based on the species certainly detected with DNA metabarcoding. Accordingly, we prorated the prey abundances of these levels proportionally into each re‐assigned species level according to the DNA metabarcoding OTU read percentages higher than the 10th percentile, within the contents of each sampled stomach; this way, we prioritized the assignments based on the intensity of the signal (i.e., OTU read percentages), giving more “weight” of the prorating to those species with a lower uncertainty, and less “weight” to those with very little signal obtained from DNA metabarcoding. To determine the weight of each prey group, we used length−weight conversion equations based on average total length of the same taxa published by Bachiller and Irigoien (2013) for the Bay of Biscay. For missing prey species or groups (i.e., observed only in the Mediterranean Sea), we assigned biomass of the same genera or the corresponding upper taxonomic level. To exclude the effect of the sample size on the identified prey abundance, we weighed the biomass of each prey group by the number of fish. We present the list of species assigned into different prey groups and/or levels, as well as the assigned weight value to each species in Table S2.

We assessed prey diversity in the diet composition with the Shannon diversity index (H′) calculated for the two mentioned (i.e., microscope‐based and corrected) mesozooplankton characterizations in stomach contents.

To ease later interpretation of the figures, we categorized prey groups into 10 groups (Table S2): Calanoids, Cyclopoids, Harpacticoids, Euphausiacea ord., Decapoda ord., Other Malacostraca, Mollusca ph., Cladocerans, Actinopterygii cl., and others (including remaining prey groups with a frequency in number <5% of the total prey consumption observed under the microscope).

For further analyses, we broke down the “Others” group in detail based on occurrence percentages observed from the DNA metabarcoding (i.e., “Others” in Table S2). We determined 54 taxonomic groups within this group, merged into 9 phyla (Table S2).

Regarding diatom (Bacillariophyta ph.) content in stomach contents, also assessed by the DNA metabarcoding, we detected 52 different algae taxa, merged into 11 groups (i.e., based on family taxonomic level, and presenting algae with <5% of the total algae occurrence as “Diatom remains” for graphical representation).

#### 2.5.2 Feeding strategy

In order to describe the diet in terms of prey importance (Bacha & Amara, 2009) and feeding strategy (specialized or generalized), we assessed prey composition following Costello (1990) graphical method using the modification by Amundsen et al. (1996), using both prey numbers (Scharf et al., 2000) and weights (Bachiller & Irigoien, 2015). We compared the percentage of non‐empty stomach in which a certain prey occurred (i.e., relative frequency of occurrence) with the percentage of abundance (frequency in numbers) and prey biomass (the weight of a particular prey item as a proportion of the total weight of all prey items in the entire stomachs). We represented the lowest identified taxonomic level in graphs, excluding prey groups with percentages of occurrence and weight <7% to ease the interpretation of the figures.

We examined patterns of relative prey size used among different size ranges of fish by generating relative frequency histograms of predator/prey size ratios (PPSRs) and biomass ratios (PPBRs) for the prey consumed, according to the methodology used by Bachiller and Irigoien (2013). A high PPSR or PPBR value indicates relatively smaller prey items ingested, whereas low values correspond to relatively larger items in stomach contents (Scharf et al., 2000).

#### 2.5.3 Inter‐specific diet overlap

We assessed the overlap in diet composition between different fish species in terms of diet dissimilarity. We used Bayesian marginal analyses considering the detailed (visual) stomach content characterization (i.e., 46 prey groups categorization) in order to define the relative diet dissimilarity index (RDI) as in Ramsey and Marsh (1984). Diet dissimilarity represents the average logit score for correctly classifying the predator species observing the diet composition; higher dissimilarity indicating higher difference between two predator species. Accordingly, if a prey taxon has a large data factor describing the role of the prey taxa in distinguishing two predator species, its presence in a diet vector strongly suggests the predator's identification (Ramsey & Marsh, 1984). This way, when comparing two predators, a small specific dissimilarity value for a specific prey item would indicate that both predators are consuming such a prey; in contrast, if a prey item shows a high dissimilarity value, its presence would be strongly related to the diet of one of the compared predator species but not the diet of the other.

The concept of “change in prey composition” or “how different the diets of each species are” may apparently seem straightforward, but there are two potential ways in which species’ diet can be “different.” Following the approach proposed by Baselga (2010, 2012), we used DNA metabarcoding presence/absence data to compute the monotonic transformation (Chao et al., 2012) of beta‐diversity—Sørensen dissimilarity index (*β*_SOR_) and its partition into two additive components, accounting for pure spatial turnover and nestedness. The turnover component is prey replacement, consists of the substitution of preys in one individual by different preys in the other individuals, and is measured as the Simpson‐based dissimilarity (*β*_SIM_). The nestedness‐resultant dissimilarity component (*β*_NES_) is prey species loss (or gain), which implies the elimination (or addition) of preys in only one individual, and leads to the poorest assemblage being a strict subset of the richest one (Baselga, 2010, 2012).

#### 2.5.4 Feeding intensity

We assessed feeding intensity calculating the stomach filling degree (SFD). This parameter allows determining if feeding intensity (or efficiency) is relatively higher, for instance, in a certain fish length range (Bachiller & Irigoien, 2015; Bachiller et al., 2016). In order to exclude the effect of fish size, the response variable SFD was defined as:

(2) $$\text{SFD}=\frac{\sum W_{p}}{\text{TL}}$$

where *W*_p_ is the weight of each prey observed in a stomach (mg) and TL is the total length of fish (mm). In order to test inter‐specific differences on SFD, we first fitted linear regressions to the total length of fish (TL) separately for each species. Then, we implemented generalized linear models (GLMs) to test the influences of TL and species on the SFD.

#### 2.5.5 Parasites and plastics

In order to test if there was any relationship among the parasite, plastic, copepods, and krill abundances (both in weight and in numbers), we computed the pairwise Spearman correlation for each species variables. We tested correlation to be significant using a level of *α* = 0.05. We also generated supplementary network plots of the correlation matrices, determining the proximity of the points with multidimensional clustering (Kuhn et al., 2020).

### 2.6 Statistical packages and plots

We used the R software v.3.6.3 (R Core Team, 2021) for all analyses and graphical representations (except Figure 1, see corresponding legend). For diet composition figures, we used the package “ggplot2” v.3.2.1 (Wickham, 2009). We calculated the isotopic SEAs and their overlap using SIBER package (Jackson et al., 2011). For the beta‐diversity analysis, we used the “betapart” package v.1.5.2 and in particular, the *betapart*.*core()* function to compute and plot diet dissimilarity, turnover, and nestedness components. In the parasites and plastic pollution analysis, we tested correlation among variables using “corrplot” package (Wei et al., 2017). We obtained supplementary network plots of correlation matrices with the *network_plot()* function of the “corrr” package (Kuhn et al., 2020). OTU table‐based matrices were directly imported into R (R Core Team, 2021).

## 3 RESULTS

### 3.1 Stable isotopes of carbon and nitrogen

Considering the longer timeframe of the diet composition, stable isotope analyses showed similar mean values of δ^13^C ranging from −19.51 to −17.85‰ in the three species. Values of δ^15^N ranged from 7.17 to 9.09‰, with sardinella having the lowest mean δ^15^N values (Table 2).

**TABLE 2:** Summary of stable isotope data for the three studied species

Species	*n*	Min–max TL	Mean δ^13^C ± SD	Min–max		Mean δ^15^N ± SD	Min–max	
*S. aurita*	17	20.2–27.2	−18.92 ± 0.30	−19.51	−18.46	8.08 ± 0.49	7.17	9.09
*E. encrasicolus*	16	13.5–16.2	−18.85 ± 0.30	−19.17	−17.94	8.11 ± 0.24	7.74	8.50
*S. pilchardus*	15	13.4–17.7	−18.99 ± 0.56	−19.49	−17.85	8.49 ± 0.22	8.10	8.91

Note

Min and Max mean minimum and maximum values, respectively.

Abbreviation: SD, standard deviation; TL, total length of fish (cm).

We found a positive and significant relationship between δ^13^C and total length in anchovy (*F*_1,14_ = 8.332, *p*‐value = .012, *R* ^2 ^= .33) and sardinella (*F*_1,15_ = 6.402, *p*‐value = .02, *R* ^2 ^= .25), while for δ^15^N only a negative and significant relationship was found for sardine (*F*_1,13_ = 7.828, *p*‐value = .0151, *R* ^2 ^= .3278) (Figure S1).

**FIGURE 2:**
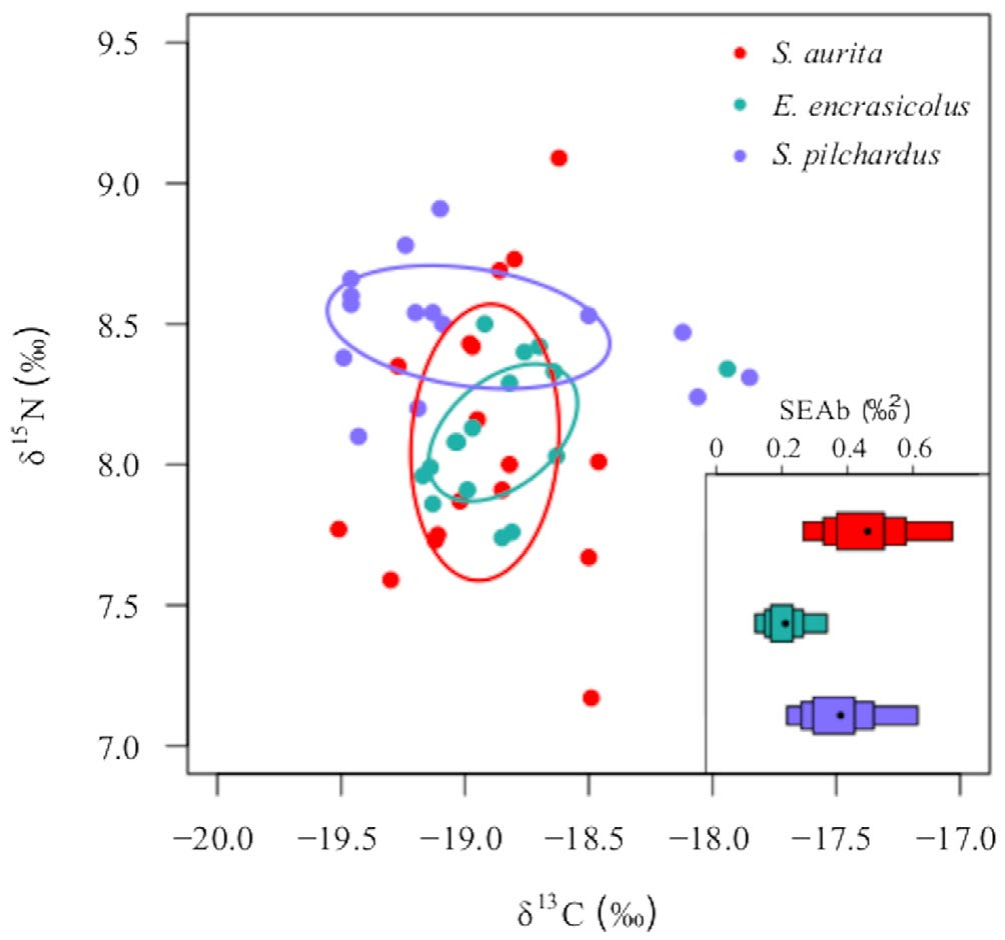
Standard ellipses area (SEA_C_) for sardinella (*Sardinella aurita*), anchovy (*Engraulis encrasicolus*), and sardine (*Sardina pilchardus*). Within the inlet, the isotopic niche width (SEA_B_) is shown

Isotopic niche was the widest for sardinella (0.46‰^2^ [0.39–0.55]), followed by sardine (0.38‰^2^ [0.32–0.46]), and then anchovy (0.21‰^2^ [0.18–0.25]) (Figure 2; Table S3a). Isotopic niche overlap was higher between sardinella and anchovy (21.00% [0–47.94] to 44.81% [0–94.91]) than with sardine (20.30% [0–49.97] to 26.37% [0–61.51]) (Table S3b).

### 3.2 Prey composition

**FIGURE 3:**
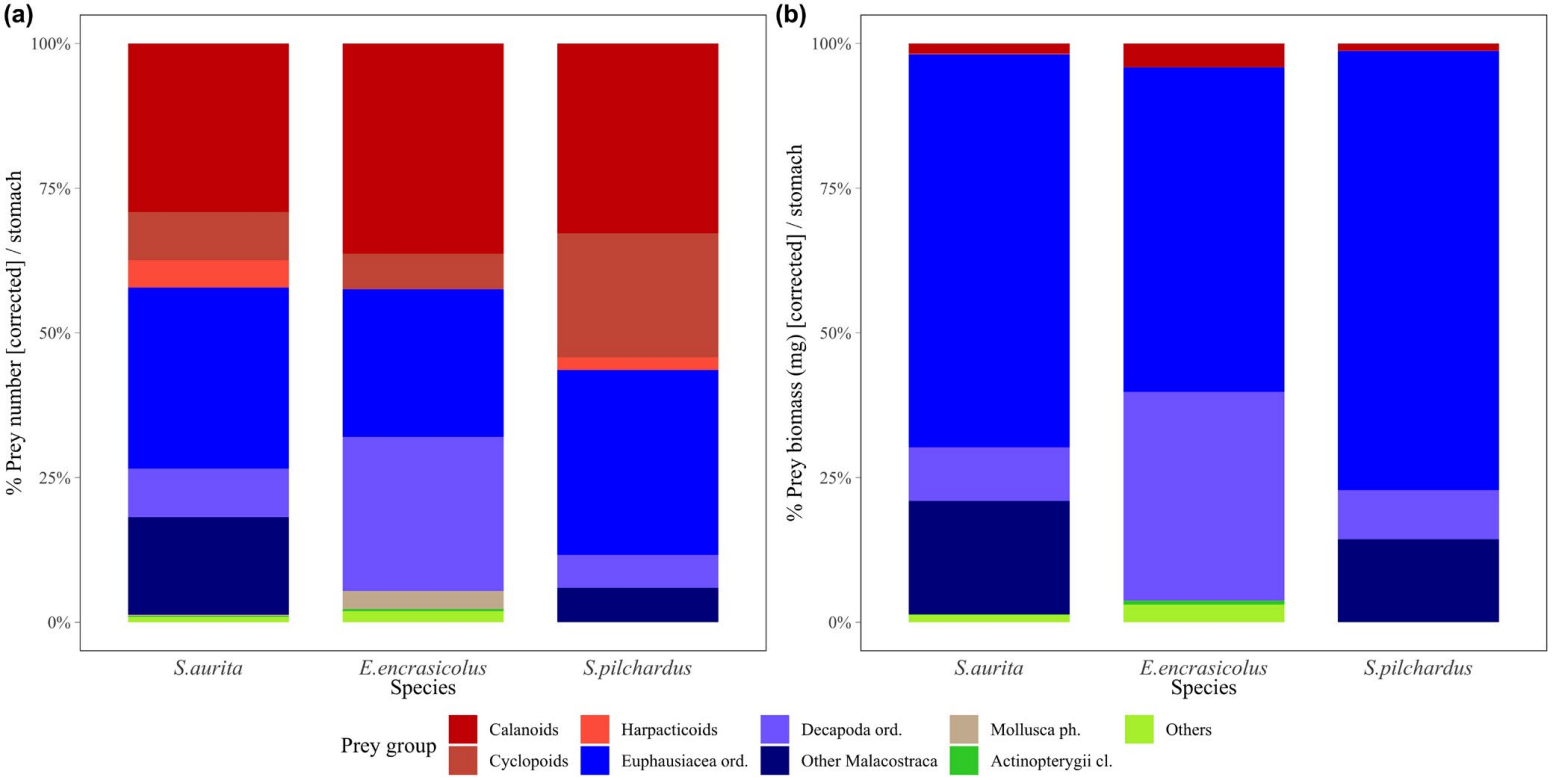
Prey group composition from stomach content analysis, as percentage of (a) mean prey abundance and (b) of mean prey biomass (mg) per stomach, for sardinella (*Sardinella aurita*), anchovy (*Engraulis encrasicolus*), and sardine (*Sardina pilchardus*), based on the corrected diet characterization

We present the detailed diet characterization with and without corrections in prey classification (see Methods) as Supplementary Material (Table S4). Euphausiids as well as other Malacostraca (including mysids and unidentified larvae and/or naupli) dominate sardinella and anchovy diet, comprising around 60% of their total prey ingestion in numbers (Figure 3a). Their diet was completed mostly with copepods and with much less frequent other prey. The diet of sardine was predominately copepods, comprising more than 50% of the prey abundance, followed by krill (32%). Regarding prey weight, the three species obtained more than 97% of the total ingested prey biomass from large prey (i.e., euphausiids, decapods, and other malacostracans; Figure 3b).

**FIGURE 4:**
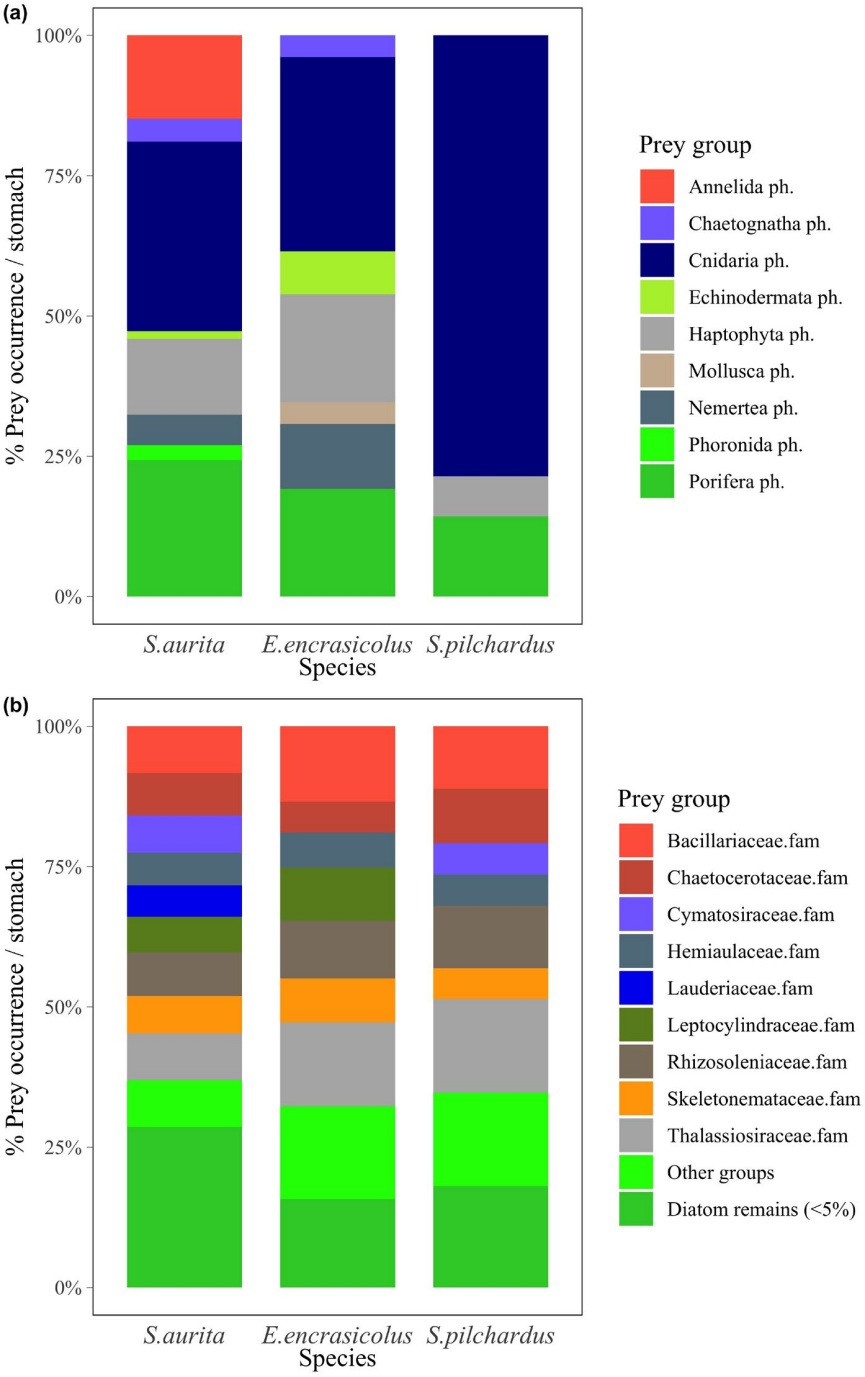
Occurrence percentage of (a) phyla within “Others” prey group and (b) diatoms, determined with DNA metabarcoding and presented for sardinella (*Sardinella aurita*), anchovy (*Engraulis encrasicolus*), and sardine (*Sardina pilchardus*)

DNA metabarcoding, despite only providing presence–absence information about prey (Amundsen & Sánchez‐Hernández, 2019), allows for detecting a wide diversity of additional prey groups that may be more easily digestible, and therefore cannot be classified with traditional microscope identification, such as annelids in sardinella or sponges (Porifera ph.), coccolithophores (Haptophyta ph.), and cnidarians in the three species. The latter group of jellyfish supposed the main prey group in terms of occurrence of these remaining groups (“others” in Table S2, Figure 4a) not determined in detail with traditional (i.e., microscope) visual diet characterization.

In addition, a wide variety of diatom groups were commonly identified by DNA metabarcoding in stomach contents of the three species, with no clear dominant species in terms of occurrence (Figure 4b). We frequently detected some species in stomach contents that are potentially harmful (Vila & Masó, 2005), such as *Pseudo*‐*nitschia delicatissima*, *P*. *galaxiae*, or *Chaetoceros socialis*, especially in sardinella (detected in >80% of samples). We present details of diatom algae species’ occurrence percentages in fish in Table S5.

Considering mesozooplankton, sardinella ingested the highest number of different prey species, therefore showing the highest Shannon diversity index, with a mean (and standard error) value of 2.31 (± 0.16); the value obtained for sardine was 1.84 (± 0.16), and anchovy showed a higher degree of specialization and therefore the lowest diversity, 1.51 (± 0.20).

### 3.3 Feeding strategy

We observed an important opportunistic predation on krill by the three predator species, as prey groups such as euphausiids and decapods appeared in high numbers (i.e., abundance) but not so frequently (i.e., low occurrence) (Figure S2; Table S4). Accordingly, euphausiid *Nematoscelis megalops* contributed to the major biomass ingested by the three species, as well as euphausiid *Euphausia krohni* in the case of anchovy and sardine, and decapod *Philocheras bispinosus* in the case of sardinella. On the other hand, the smallest prey size range, including calanoids (e.g., *Candacia armata*, *Diaixis Hibernica*, *Paracalanus parvus*, and *Bradyidius armatus*) and cyclopoids (e.g., *Oncaea* spp.), also appeared as an important food resource in the diet of the three species, suggesting a generalized complementary filter‐feeding behavior, despite being less relevant in terms of biomass input in the diet (Figure S2).

**FIGURE 5:**
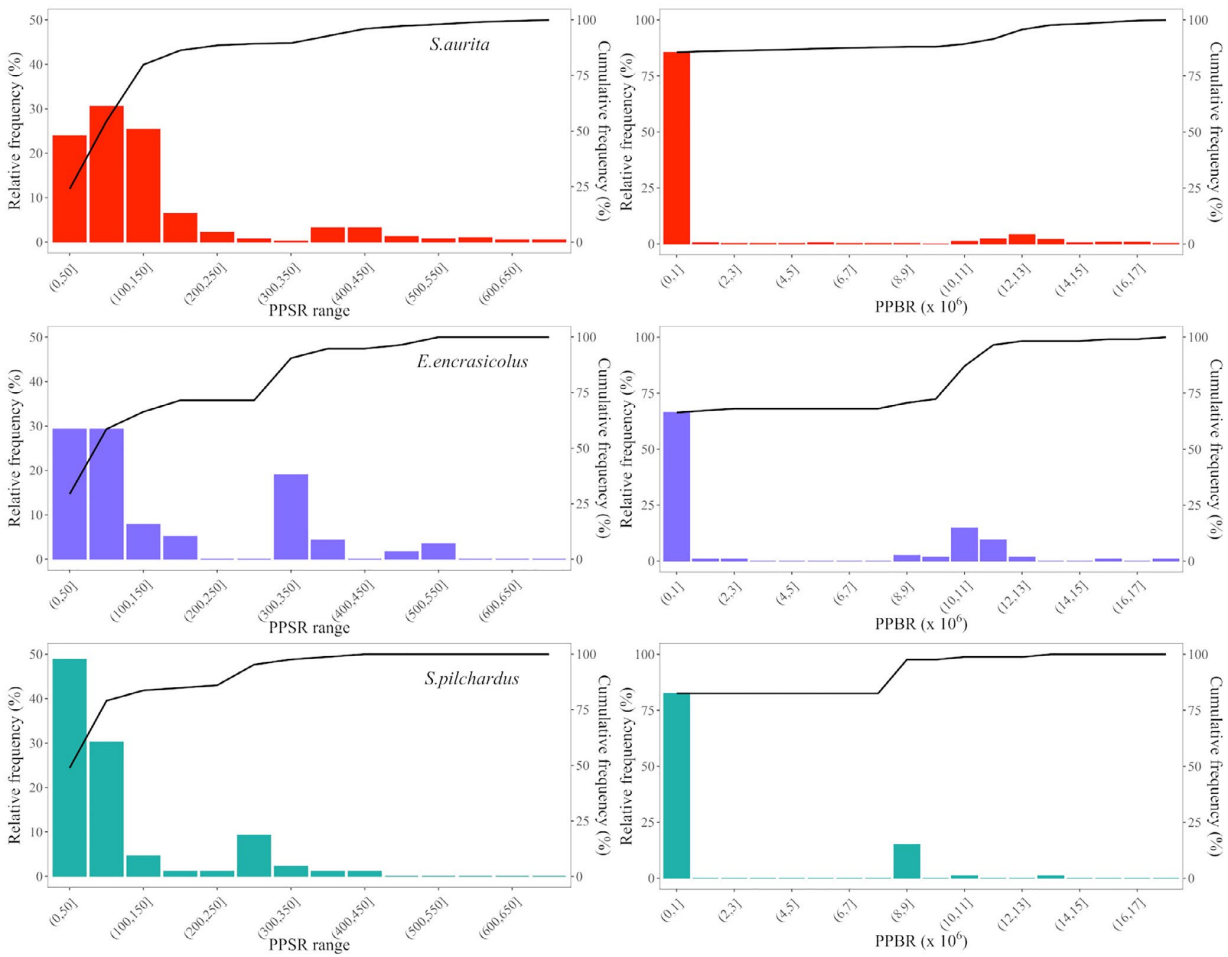
Relative frequency distribution of predator–prey size ratios (PPSR, left side) and predator–prey biomass ratios (PPBR, right side) in the three fish species: sardinella (*Sardinella aurita*), anchovy (*Engraulis encrasicolus*), and sardine (*Sardina pilchardus*), based on the corrected diet characterization. Cumulative PPSR and PPBR frequencies are indicated by continuous lines (note the secondary *Y* axis)

Regarding the predator/prey ratios (PPSR and PPBR; Figure 5), relatively large prey composed more than the 50% of the total prey size range in the diet of the three species, and more than the 60% of the total prey biomass ingestion (>75% for sardinella and sardine). In relation to fish size, the largest prey (i.e., euphausiids and decapods) were especially important in numbers in the diet of sardine, whereas sardinella, and especially anchovy, completed their diet ingesting relatively smaller prey (i.e., copepods) in higher numbers. In any case, the three predator species showed a similar prey size frequency distribution (PPSR) and an almost equal prey biomass frequency distribution (PPBR) in relation to their body size. PPBR distribution in sardinella highlighted that >80% of the ingested biomass was derived from large prey, while the rest of the prey size distribution was distributed in different ranges, varying more than the small prey size ranges in the diet of anchovy or sardine (Figure 5).

### 3.4 Inter‐specific diet overlap

The pairwise inter‐specific comparisons in the relative diet dissimilarity (RDI) showed that anchovy and sardine were relatively the most different (mean RDI = 0.84), whereas sardinella showed lower values (i.e., relatively more similar diet composition) when compared with anchovy (mean RDI = 0.76) and sardine (mean RDI = 0.79). Regarding specific dissimilarity indices (Figure S3), most of the decapod and copepod species were randomly related to the diet of the three species (e.g., *Galathea* sp. and *Paracalanus parvus* in sardinella, *Anapagurus chiroacanthus* and *Bradyidius armatus* in sardine, and *Philocheras bispinosus* and *Clausocalanus* spp. in anchovy), without showing any clear trends on specific prey groups related to certain predator species. This way, euphausiids like *Meganyctiphanes norvegica* and *Nematoscelis megalops* seemed to be more related to the diet of anchovy, whereas *Euphausia krohni* and euphausiid (i.e., Capyptopis and Furcilia) larvae occurred more frequently in the diet of sardine. In any case, the main inter‐specific differences were due to other prey groups such as siphonophores *Muggiaea atlantica* and *Abylopsis tetragona* or jellyfish *Leuckartiara octona*, which were more related to the diet of sardinella than to the diet of anchovy or sardine (Figure S3).

**FIGURE 6:**
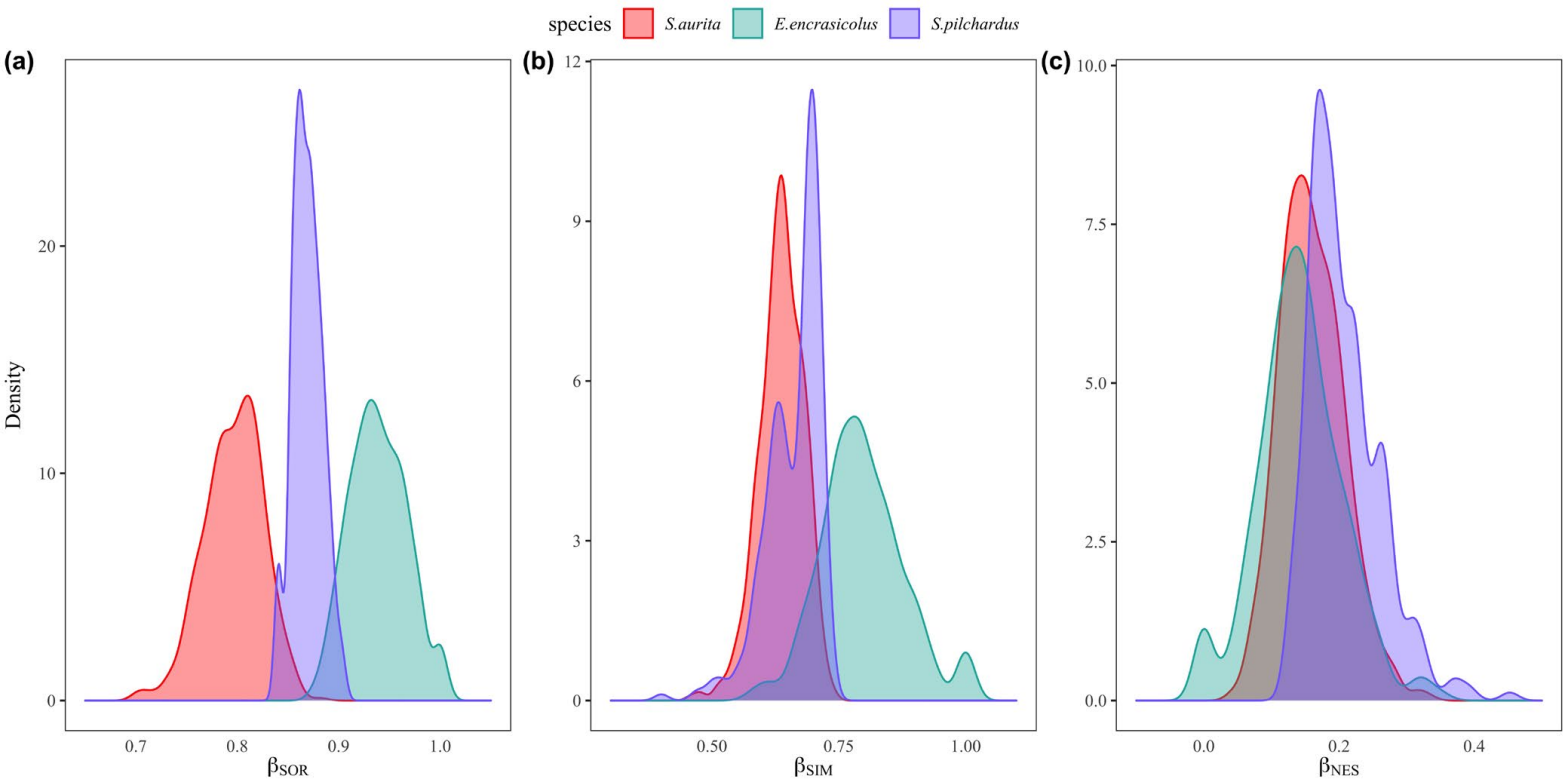
The partition of beta‐diversity transformed as (a) Sørensen dissimilarity index (*β*_SOR_), and the partition in the (b) turnover (*β*_SIM_) and (c) nestedness (*β*_NES_) components, for sardinella (*Sardinella aurita*, red), anchovy (*Engraulis encrasicolus*, light green), and sardine (*Sardina pilchardus*, blue)

Regarding variations in prey composition (i.e., beta‐diversity), the Sørensen dissimilarity index (*β*_SOR_) was higher for anchovy than for the other species, whereas sardinella showed the lowest individual variations in prey composition (Figure 6a). The higher beta‐diversity observed in anchovy was primarily explained by the turnover component (i.e., Simpson dissimilarity, *β*_SIM_), which indicated that anchovy was the most different species (i.e., the most variable predator in terms of prey composition) due to a higher prey replacement in comparison with sardine and sardinella (Figure 6b). The nestedness component (*β*_NES_) was similar for the three species, which means that the studied fish individuals had a similar prey loss (i.e., prey richness) (Figure 6c).

### 3.5 Feeding intensity

Feeding intensity (stomach filling degree, SFD) was significantly higher with growth in sardinella, whereas in anchovy and sardine the feeding intensity decreased with fish size (total length of fish, TL) (Figure S4). The applied log‐link Poisson regression GLM (Shapiro–Wilk normality tests; sardinella: *W* = 0.73, *p* < .001; anchovy: *W* = 0.91, *p* < .001; and sardine: *W* = 0.89, *p* < .001) showed that inter‐specific differences on SFD were also statistically significant (Chi‐square test *p *< .001 for SFD vs. TL, SFD vs. species. and SFD vs. TL*species).

### 3.6 Parasites and plastics

**FIGURE 7:**
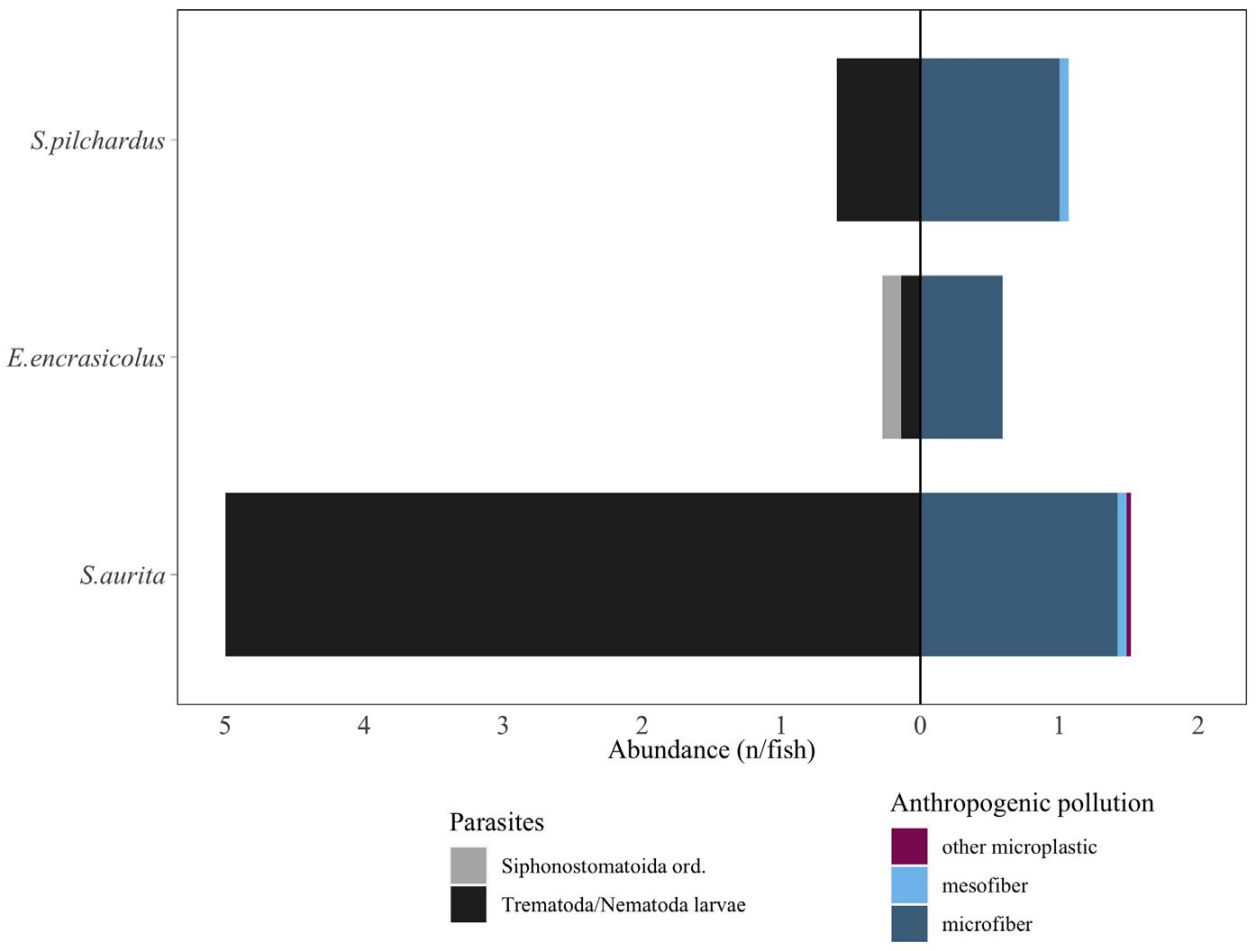
Mean abundance of parasites in stomach contents (left side of the plot, grey scale) and mean anthropogenic fiber ingestion (right side of the plot, blue–purple scale), classified by type and weighed by the total number of sampled fish

We found anthropogenic fibers and parasites in stomach contents of the three species (Figure 7). Parasites were mostly trematod and/or nematod larvae, with siphonostomatoid copepods also found in few anchovies. Most anthropogenic items were <2 mm microfibers, whereas mesofibers and other items like plates (e.g., paint and/or plastic remains) appeared in low numbers. Stomach contents of sardinella showed higher abundance of anthropogenic fibers in comparison with the other two species, with more than the double of microfibers than those ingested by anchovies, which showed the lowest degree of anthropogenic pollution. This was in apparent relation with the number of parasites found in stomach contents, considering that sardinella showed much higher abundance of trematoda and/or nematod larvae in comparison with the other two species (Figure 7). Nevertheless, we did not find any significant relationship between parasitic organisms found in stomachs and total prey abundance, nor with anthropogenic pollution in stomach contents (network Spearman correlation plots are presented in Figure S5).

### 3.7 Methodological assessment

The corrected diet characterization (i.e., applying the taxonomic re‐assignment of uncertain prey items determined under the microscope after checking DNA metabarcoding data) often resulted in higher prey number, biomass, and occurrence frequencies of (especially) calanoid copepods and decapods, if compared with the values obtained based only on the microscope analyses of the three fish species (Figure S6a). In contrast, some other groups could have also been underestimated if only DNA metabarcoding data would have been considered, since many cladocerans, mollusks, or fish eggs (Actynopterygii cl.) were certainly counted and identified under the microscope, but rarely detected with genetics. In addition, we reclassified prey items classified under the microscope within more general or uncertain groups (i.e., with relatively lower taxonomic detail), such as “other malacostraca,” into other verified taxa determined by the corrected diet characterization (e.g., as decapods or euphausiids). In the same way, the higher numbers of certified krill (i.e., large prey) determined to their corresponding taxonomic classification resulted in lower PPSR and PPBR ratio estimates when applying the correction (i.e., combination of methods), especially in the case of sardinella and anchovy (Figure S6b). More taxa of small prey determined with DNA metabarcoding increased the diversity, especially in the diet of sardinella (Figure S6b), which also resulted in higher estimates of small prey biomass (i.e., high PPBR ranges; Figure S7).

The mean diet dissimilarity values increased slightly when the corrected prey classification was applied, since higher detail in prey classification resulted in higher differences between diet compositions (Figure S6c).

## 4 DISCUSSION

In this study, we used three complementary methodologies to analyze the potential trophic overlap of the round sardinella with two sympatric, economically important species, anchovy and sardine, in the Western Mediterranean Sea. Combining novel methods (DNA metabarcoding) with classic approaches (visual stomach content characterization and stable isotope analyses of carbon (δ^13^C) and nitrogen (δ^15^N)), the obtained diet characterization provides new relevant information; not only considering the main groups normally observed by visual analysis (e.g., copepods and krill) but also detecting other groups like jellyfish and diatoms, which would have been underestimated without DNA analysis (Albo‐Puigserver et al., 2019, and references therein).

The higher number of prey species often detected in DNA metabarcoding improved the determination of trophic overlap and niche width observed from visual analysis. Also, isotopic niche width from δ^13^C and δ^15^N values in muscle, which integrates a longer trophic timeframe, allowed checking whether the overlap detected with these techniques is conserved in a longer timescale. Although the combination of different techniques needs a higher effort on sample analysis that could suggest some degree of sample size limitation, our sample coverage was representative of the diet composition of the three species (Figure S8). However, the three techniques show biases. For example, microscope analysis is obviously limited to the taxonomic resolution, as well as to the digestion state of the stomach contents (i.e., due to preservation and/or time between the ingestion and the sampling). On the other hand, the ability to detect much higher number of taxa from digested prey remains by DNA metabarcoding can result in prey richness overestimation (Sakaguchi et al., 2017). In addition, prey detection observed under the microscope (i.e., verified) but not determined by DNA metabarcoding could be the consequence of both DNA degradation (Kress et al., 2015) and primer performance, due to differential amplification of the prey species or to the specificity of the blocking primer. Although specifically designed to prevent DNA amplification for the three species under study, DNA from other fish species could also have been blocked (Piñol et al., 2015). In any case, the correction method applied in this study overcomes the limitation of presence–absence‐based DNA metabarcoding information in diet studies (Amundsen et al., 1996; Riccioni et al., 2018) and could be useful for further developments in detailed diet characterization studies with other species. Finally, stable isotope analysis of our adult fish muscle integrates trophic information on a longer timescale, but without taxonomic resolution and with a temporal resolution of few months. This way, analyses of fish fin might also offer a longer timeframe (Navarro et al., 2020), but would not be comparable to stomach content analyses due to the lack of juvenile samples. According to that, it is when combining all three methods that trophic information is more complete for characterizing the diet, as well as trophic niche overlap within the Gulf of Alicante, an area with similar oceanographic conditions (García‐Rodríguez et al., 2021), that allow making diet comparisons over short and long timeframes.

From an ecological point of view, our results confirm a generalist diet in the three species in the Western Mediterranean (e.g., Albo‐Puigserver et al., 2019; Bachiller et al., 2020), with an important opportunistic active predation upon larger prey which completed their diet with continuous filter feeding activity ingesting mainly calanoid copepods (Table 3). Large prey are of special interest, since their ingestion contributes for a better body condition of the fish (Queiros et al., 2019) and they also play a different role in energy transfer within the food web (Heneghan et al., 2016). In line with Bachiller et al. (2020), both anchovy and sardine show a higher relative biomass income from krill, especially euphausiids, with less ingestion on copepods and other prey (e.g., cladocerans or fish eggs). Still, also from our study, sardine maintains higher copepod ingestion than anchovy. Sardinella, in accordance with its high feeding plasticity and adaptability to environmental fluctuations (Albo‐Puigserver et al., 2019; Morote et al., 2008; Tsikliras, 2008), seem to be an even more effective feeder on large prey, such as decapods and euphausiids, than anchovy and sardine (Table 3). Our results confirm a prey preference upon krill, not reported in previous studies (Karachle & Stergiou, 2017; Lomiri et al., 2008; Madkour, 2012; Tsikliras et al., 2005).

**TABLE 3:** Conceptual summary table containing the main results obtained with different methodological approaches for sardinella (*Sardinella aurita*), anchovy (*Engraulis encrasicolus*), and sardine (*Sardina pilchardus*)

Methodological approach	Variable	Species		
		*S. aurita*	*E. encrasicolus*	*S. pilchardus*
Stable isotopes δ^13^C and δ^15^N	δ^13^C–TL relationship	Positive^a^	Positive^a^	(ns)
	δ^15^N–TL relationship	(ns)	(ns)	Negative^a^
	Isotopic niche area	Widest 0.46‰^2^	0.21‰^2^	0.38‰^2^
	Isotopic niche overlap	*S*. *aurita*–*E*. *encrasicolus* > *S. aurita*–*S*. *pilchardus*		
Stomach contents (visual analysis)	SFD–TL relationship	Positive^a^	Negative^a^	Negative^a^
	Parasites	Highest	Lowest	
	Anthropogenic pollution	Highest	Lowest	
Stomach contents corrected by DNA metabarcoding	Prey number (main contribution)	euph./decap./malac. (highest abundance)	euph./decap./malac.	cal.cop./euph.
	Prey biomass (main contribution)	euph./decap./malac.	euph./decap./malac.	euph./decap./malac.
	Shannon diversity index	2.31	1.51	1.84
	PPSR	>75% large prey	~60% large prey	>75% large prey
	PPBR	>80% large prey	~60% large prey	>75% large prey
	Relative diet dissimilarity	Highest difference in jelly/siph.	Highest RDI between *E*. *Encrasicolus–S*. *pilchardus*	
	Beta‐diversity	Lowest individual variation in prey composition	Most different due to higher prey replacement (turnover)	
		Similar nestedness in the three species		

^a^ Denotes significant difference (*p* < .01); ns means not significant relationship; “cal.cop.”: calanoid copepods; “euph.”: euphausiids; “decap.”: decapods; “malac.”: malacostracans; “jelly”: jelly organisms; “siph.”: siphonophores.

On the other hand, a higher diversity of small prey in the stomach contents of sardinella, also confirmed by their wider isotopic niche width (especially in the δ^15^N axis; Table 3), suggests that this opportunistic feeder (Albo‐Puigserver et al., 2019; Tsikliras et al., 2005) with a lengthier gut (Karachle & Stergiou, 2013) is able to adapt to different feeding conditions (i.e., prey availability) more efficiently than the other two species. In fact, anchovy showed a less diverse diet composition than the other two species, corroborated by the smaller isotopic niche width, probably due to the high degree of large prey ingestion (Table 3), in spite of being able to switch to filter feeding in other conditions (Nikolioudakis et al., 2014; Tudela & Palomera, 1997). Nevertheless, inter‐specific differences regarding small prey may be due to prey availability during the continuous filter‐feeding ingestion, rather than due to any active prey selection upon certain copepods. Regarding the phytoplankton, although sardine might be considered as the most effective filter‐feeder species (Albo‐Puigserver et al., 2016, 2019; Costalago et al., 2015; Nikolioudakis et al., 2012), our results from DNA metabarcoding in combination with stomach content suggest a passive diatom ingestion when filtering the water for the three species, as part of their zooplanktivorous and/or selective predation on larger prey. However, there is also a bias here, since part of the phytoplankton observed in stomach contents might be due to remains of the diatoms ingested by herbivorous copepods included in the diet of fish (Benedetti et al., 2016; Mauchline, 1998).

Anchovy showed a larger individual variability in large prey ingestion (i.e., prey replacement), which was partly reflected with a relative higher diet dissimilarity and lower isotopic niche overlap between anchovy and sardine (Table 3). In any case, for sardinella, our results evidence its effective diet plasticity, which might have resulted also in smaller individual variation (i.e., Sørensen dissimilarity), due to the wider range of prey size and diversity, and high degree of niche overlap between sardinella compared with the other two species (Table 3). Such an overlap contrasts with the niche segregation observed for this species in 2012–2013 in the Ebro Delta (Northwestern Mediterranean Sea), where sardinella presented higher δ^15^N than anchovy and sardine (Albo‐Puigserver et al., 2019). In our more southern study area, we found similar and higher δ^15^N of sardine and anchovy compared to sardinella. This higher isotopic values of sardine and anchovy, and higher trophic overlap with sardinella in comparison to the study from the Ebro Delta area, could be related to the similar prey size we observed for the three species in our study area and supports previous findings that described a southwards increase in consumption of larger prey (i.e., decapods and euphausiids) for sardine and anchovy (Bachiller et al., 2020). It should be noted that diet dissimilarity is not mainly driven by differences in krill ingestion, but by other prey, such as siphonophores and jellyfish, frequently found not only in anchovy and sardine, in line with Bachiller et al. (2020), but also in sardinella (Table 3). Such prey could also be considered as prey types close to salps and cladocerans observed by Albo‐Puigserver et al. (2019). In fact, ingestion of jelly organisms by sardinella, or at least its detection (perhaps due to methodological developments), was only reported twice, and more than a decade ago (Lomiri et al., 2008; Tsikliras et al., 2005). Hence, the incorporation of DNA analysis as a common technique in stomach content analysis will benefit the identification of soft preys such as jellyfishes, improving the understanding of trophic relationship between different functional groups in food‐web studies.

In any case, the high degree of diet overlap between the three species highlighted by our results does not necessarily mean competition, at least if there is enough food available for species to achieve their optimum fitness (Bachiller & Irigoien, 2015). However, top‐down control of zooplankton by these planktivorous species (Checkley et al., 2009) suggests that interactions might occur when changing feeding conditions in future shifting distribution scenarios (Pennino, Coll, et al., 2020) due to the increase in sea surface temperatures in the Mediterranean Sea (Pisano et al., 2020). In fact, a northward expansion of temperate species, such as sardinella (Maynou et al., 2020; Sabatés et al., 2006; Tsikliras, 2008), can occur, which may lead to stronger inter‐specific influences. In such case, our results suggest that sardinella would be able to effectively adapt their feeding activity, for example, switching to filter feeding (i.e., including both phyto‐ and zooplankton) when krill availability is low, or obtaining their ingested biomass from other large prey like jellyfish and siphonophores as they increase in abundance, as an effect of the seawater warming (Bellido et al., 2020; Purcell, 2012). In relation to this, Bachiller et al. (2020) reported higher inter‐specific differences in sardine and anchovy, as species moved northwards, with a higher relative ingestion of microalgae (i.e., diatoms) when large prey availability decreased along an inverse latitudinal gradient (i.e., south to north).

Finally, anthropogenic marine pollution (i.e., fibers and plastics) ingestion contributed to all three fish species. While fiber ingestion was already reported for anchovy and sardine (Capone et al., 2020; Compa et al., 2018; Pennino, Bachiller, et al., 2020), our results confirm that this problem also affects sardinella (Table 3). This affection is even larger for sardinella because it has the highest feeding intensity. While not significant, we found that stomachs with higher anthropogenic fiber occurrences also showed higher abundance of parasites (Table 3), as previously observed (Pennino, Bachiller, et al., 2020). If such a relation is confirmed in further studies, it might also indicate an extra threat to pelagic fish health condition, which could be aggravated especially in the future with a potential decrease in large prey predation and therefore also in fish condition (Queiros et al., 2019).

In conclusion, the trophic niche overlap might be irrelevant for sardinella, anchovy, and sardine when food availability is high due to the ability in the three species to adapt their feeding to prey availability. However, the diet plasticity (e.g., including smallest copepods, largest krill, and even siphonophores and anthozoans) of the recently incorporated (and expanding) sardinella might increase the inter‐specific competition in the pelagic fish community, especially in potential future scenarios with more jellyfish and less large zooplankton (i.e., poorer feeding conditions). In such a case, potential negative socioeconomic effects might be relevant through changes in body condition of the highly commercial anchovy and sardine, or even fluctuations in their fisheries, that is, caused by the role of sardinella as potential competitor. The increased anthropogenic marine pollution might also lead to a higher degree of plastic ingestion, especially by sardinella, although whether this could also cause potential diseases (i.e., presence of parasites in stomachs) is still uncertain. Our results are relevant within the context of high exploitation of commercial resources in the Mediterranean Sea and the observed rate of climate change, and should inform applied studies aiming at operationalizing the ecosystem‐based approaches to fisheries management in the region.

## CONFLICT OF INTEREST

The authors declare that the research was conducted in absence of any commercial or financial relationship that could be construed as potential conflict of interest.

## AUTHOR CONTRIBUTIONS

**Eneko Bachiller:** Conceptualization (lead); Data curation (lead); Formal analysis (lead); Investigation (lead); Methodology (lead); Resources (equal); Software (equal); Supervision (lead); Validation (lead); Visualization (lead); Writing‐original draft (lead); Writing‐review & editing (lead). **Joan Giménez:** Conceptualization (equal); Data curation (supporting); Formal analysis (equal); Investigation (equal); Methodology (supporting); Resources (equal); Software (supporting); Validation (equal); Visualization (equal); Writing‐original draft (equal); Writing‐review & editing (equal). **Marta Albo‐Puigserver:** Conceptualization (equal); Data curation (supporting); Formal analysis (equal); Investigation (equal); Methodology (supporting); Resources (equal); Software (supporting); Validation (equal); Visualization (equal); Writing‐original draft (equal); Writing‐review & editing (equal). **Maria Grazia Pennino:** Data curation (supporting); Formal analysis (supporting); Methodology (supporting); Software (supporting); Writing‐review & editing (supporting). **Neus Marí‐Mena:** Data curation (supporting); Formal analysis (supporting); Methodology (supporting); Writing‐review & editing (supporting). **Antonio Esteban:** Writing‐review & editing (supporting). **Elena Lloret‐Lloret:** Visualization (supporting); Writing‐review & editing (supporting). **José María Bellido:** Conceptualization (equal); Funding acquisition (equal); Investigation (supporting); Project administration (equal); Supervision (supporting); Validation (supporting); Visualization (supporting); Writing‐review & editing (supporting). **Marta Coll:** Conceptualization (equal); Funding acquisition (equal); Investigation (supporting); Methodology (supporting); Project administration (equal); Supervision (supporting); Validation (supporting); Visualization (supporting); Writing‐original draft (supporting); Writing‐review & editing (supporting).

## Supporting information

Supplementary Material

## Data Availability

All the DNA metabarcoding raw reads of prey species obtained from the stomach contents were deposited in the BioProject database (Gen‐Bank, NCBI, 1988) as BioProject PRJNA653773. All other data used in the manuscript are included in the manuscript or as Supporting Information.
