# Supplementary material for: Trophic niche overlap between round sardinella (Sardinella aurita) and sympatric pelagic fish species in the Western Mediterranean
Source: Ecol Evol. 2021 Nov 10;11(22):16126–42. doi: 10.1002/ece3.8293 (PMC8601905; doi:10.1002/ece3.8293)
Supplement: Supplementary file 1 — Supplementary Material [file ECE3-11-16126-s001.docx]

**Trophic niche overlap between round sardinella (*Sardinella aurita*) and sympatric pelagic fish species in the Western Mediterranean**

**Eneko Bachiller^1,2,*^, Joan Giménez^1,3,4^, Marta Albo-Puigserver^1,5^, Maria Grazia Pennino^6^, Neus Marí-Mena^7^, Antonio Esteban^8^, Elena Lloret-Lloret^1^, José María Bellido^8^, Marta Coll^1^**

^1^ Marine Renewable Resources Department, Institute of Marine Science (ICM-CSIC). Barcelona, Spain.

^2^[current address]: AZTI, Sustainable Fisheries Management (Data), Basque Research and Technology Alliance (BRTA). Sukarrieta, Bizkaia (Basque Country), Spain.

^3^ MaREI Centre, Environmental Research Institute, University College Cork, Cork, Ireland.

^4^ School of Biological, Earth, and Environmental Sciences, University College Cork, Cork, Ireland.

^5^Centro de Ciências do Mar, Universidade do Algarve (CCMAR-UAlg). Faro, Portugal.

^6^Centro Oceanográfico de Vigo, Instituto Español de Oceanografía. Vigo, Spain.

^7^AllGenetics & Biology SL. Cubelos, 21, Perillo. Oleiros, A Coruña, Spain.

^8^Centro Oceanográfico de Murcia, Instituto Español de Oceanografía. San Pedro del Pinatar, Murcia, Spain.

*** Correspondence:**

ebachiller@mail.com


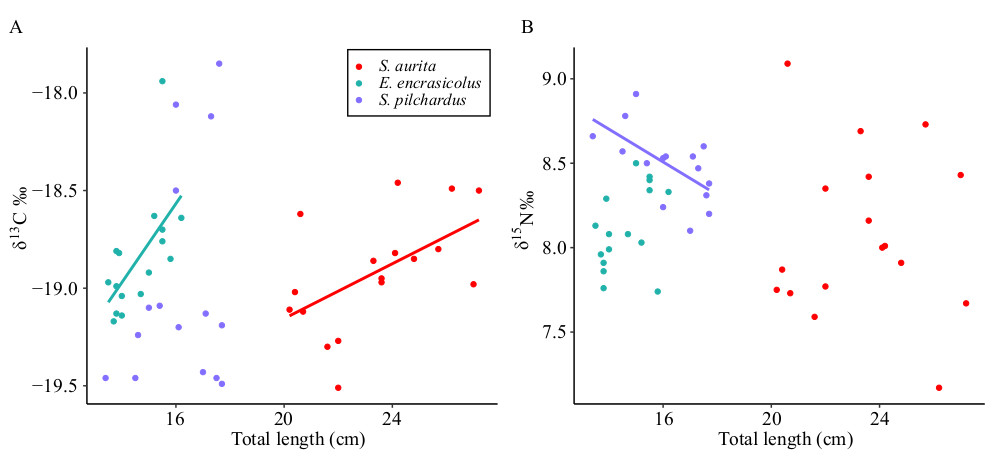


**Figure S1.** Relationship between stable isotopes values of **(a)** carbon (δ^13^C) and **(b)** nitrogen (δ^15^N) and the total length of fish (cm) in sardinella (*S. aurita*), anchovy (*E. encrasicolus*) and sardine (*S. pilchardus*).


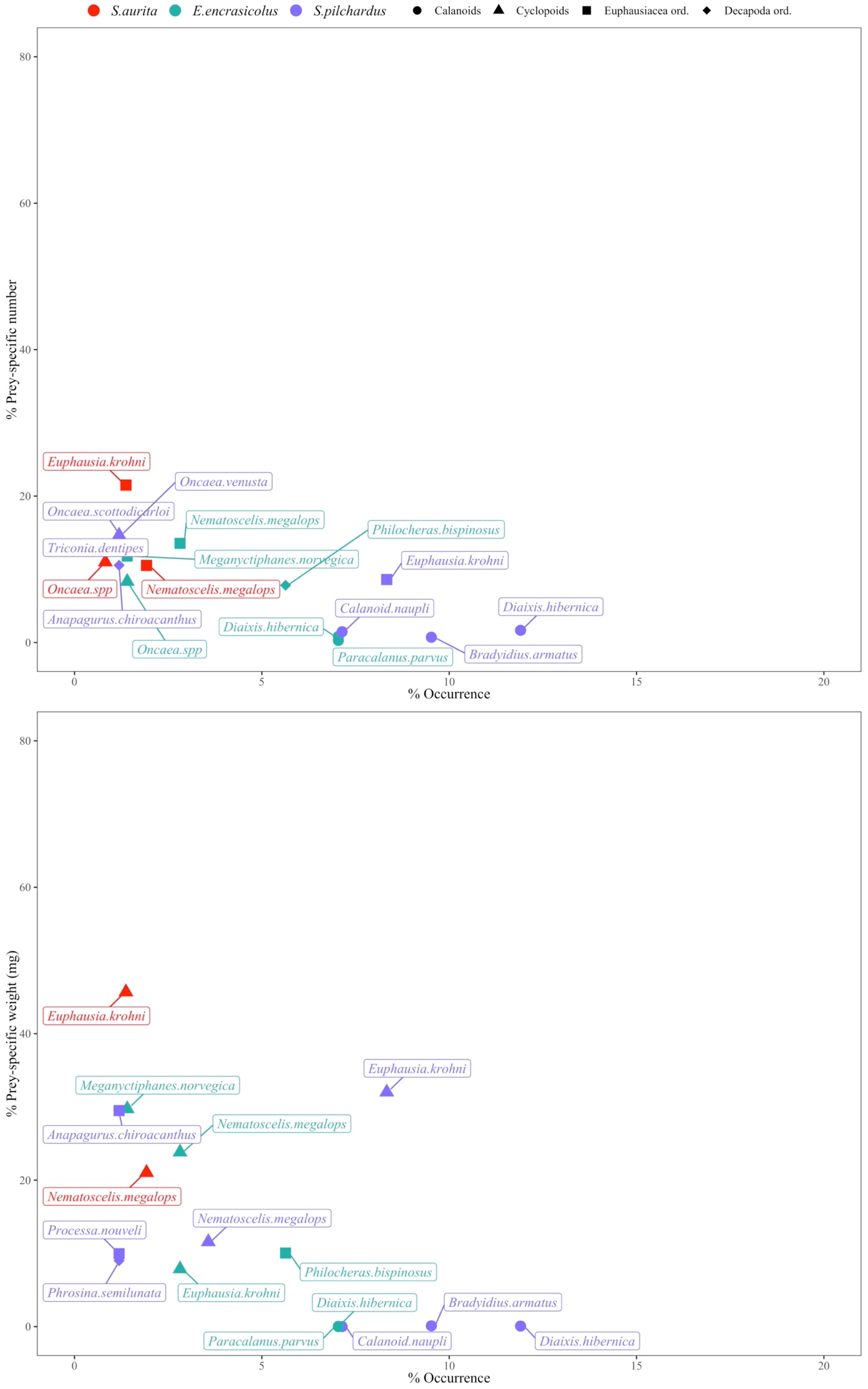
**Figure S2.** Graphical representation of dominant prey for sardinella (*S. aurita*), anchovy (*E. encrasicolus*) and sardine (*S. pilchardus*), according to Costello (1990) with modification by Amundsen *et al*. (1996), and based on the corrected diet characterization. %Occurrence is the relative frequency of occurrence of a certain prey item in stomachs, whereas %Prey-specific number and %Prey-specific weight are the abundance percentage and the percentage weight of the specific group relative to the total prey found in the stomachs in which each prey group appeared, respectively. Point shapes correspond to the 9 prey group categorization used in previous figures, and prey labels are colored based on predator species. Note different scales of X and Y axes.

**
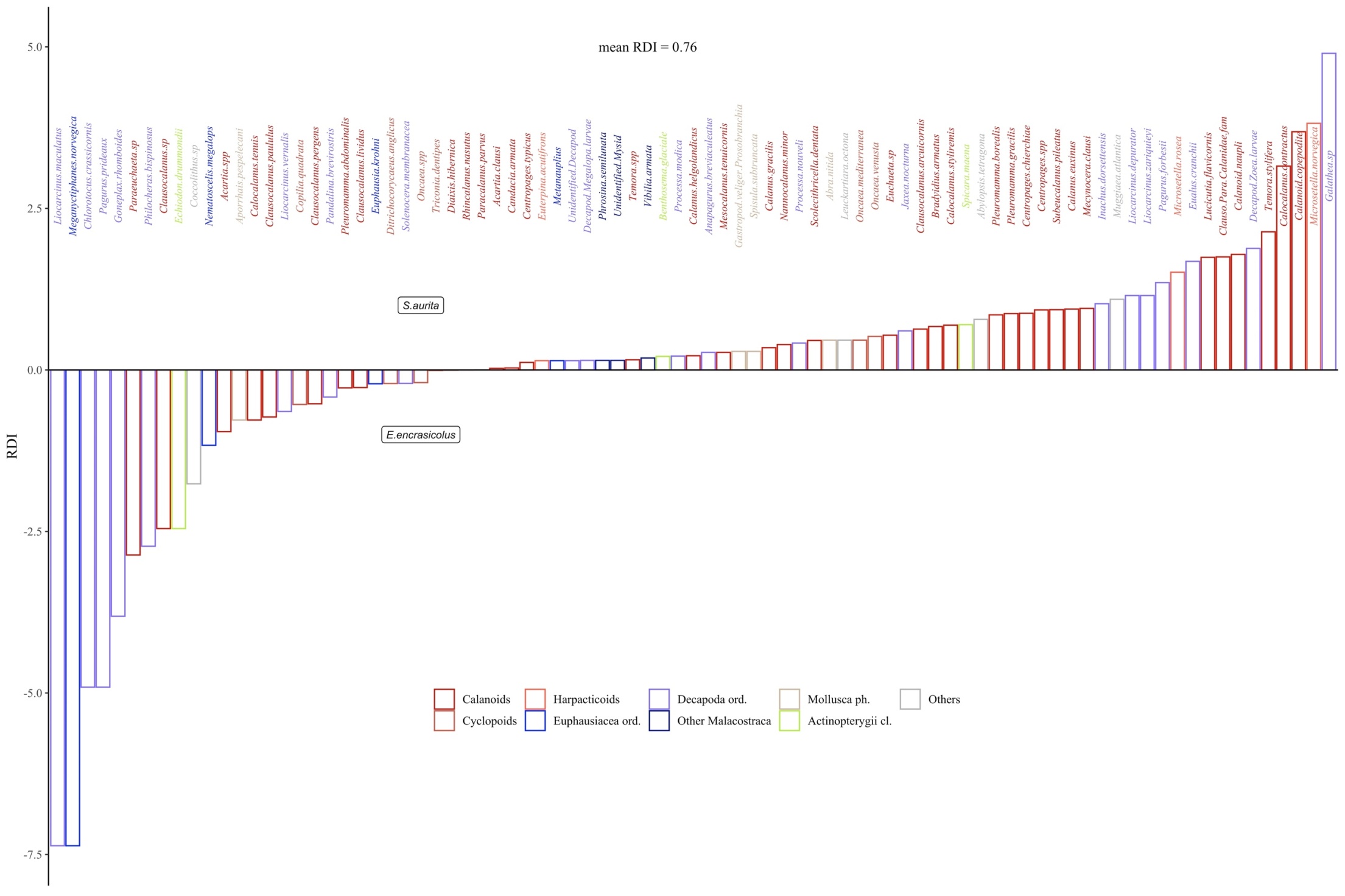
**

**
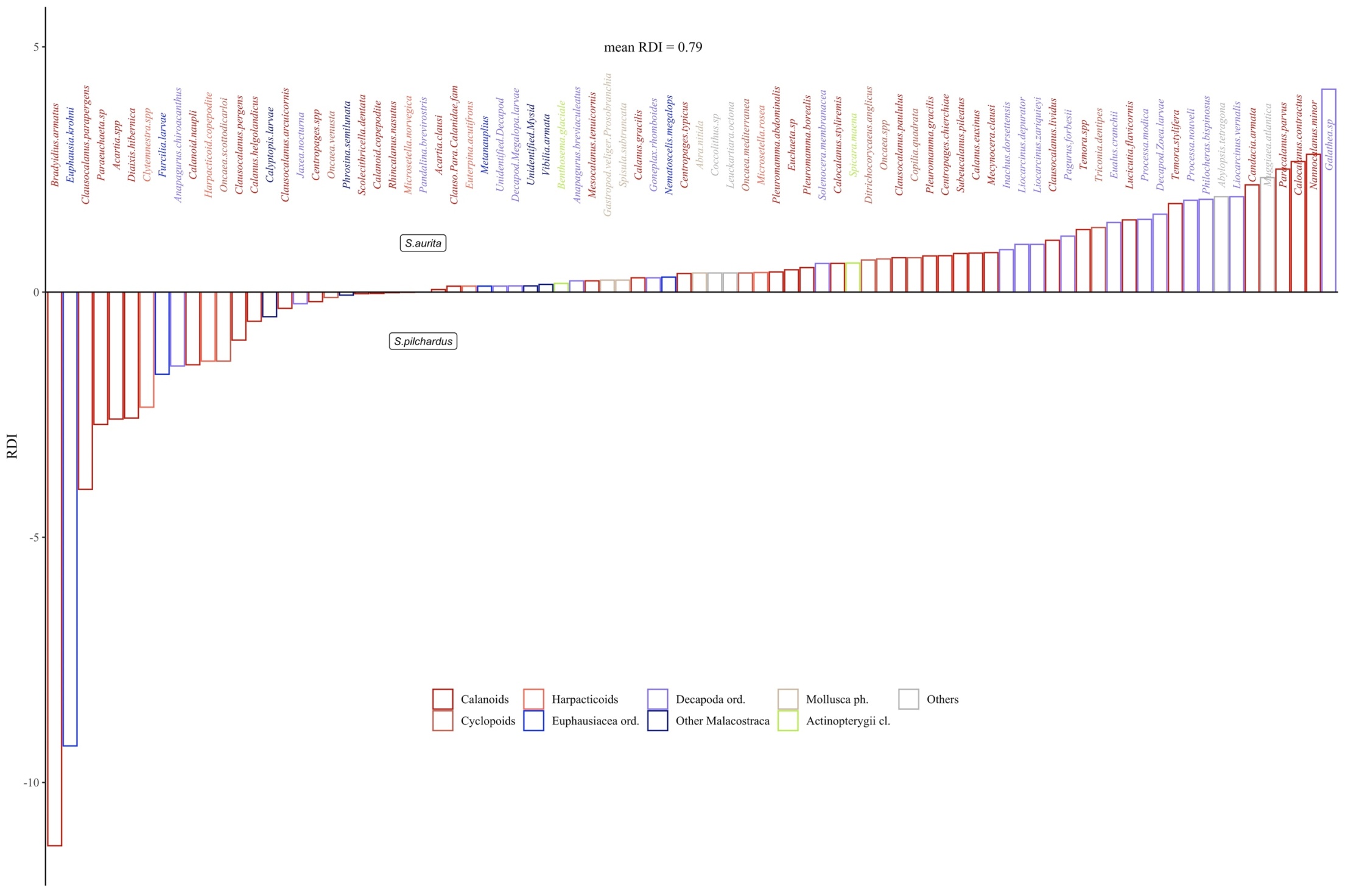
**

**
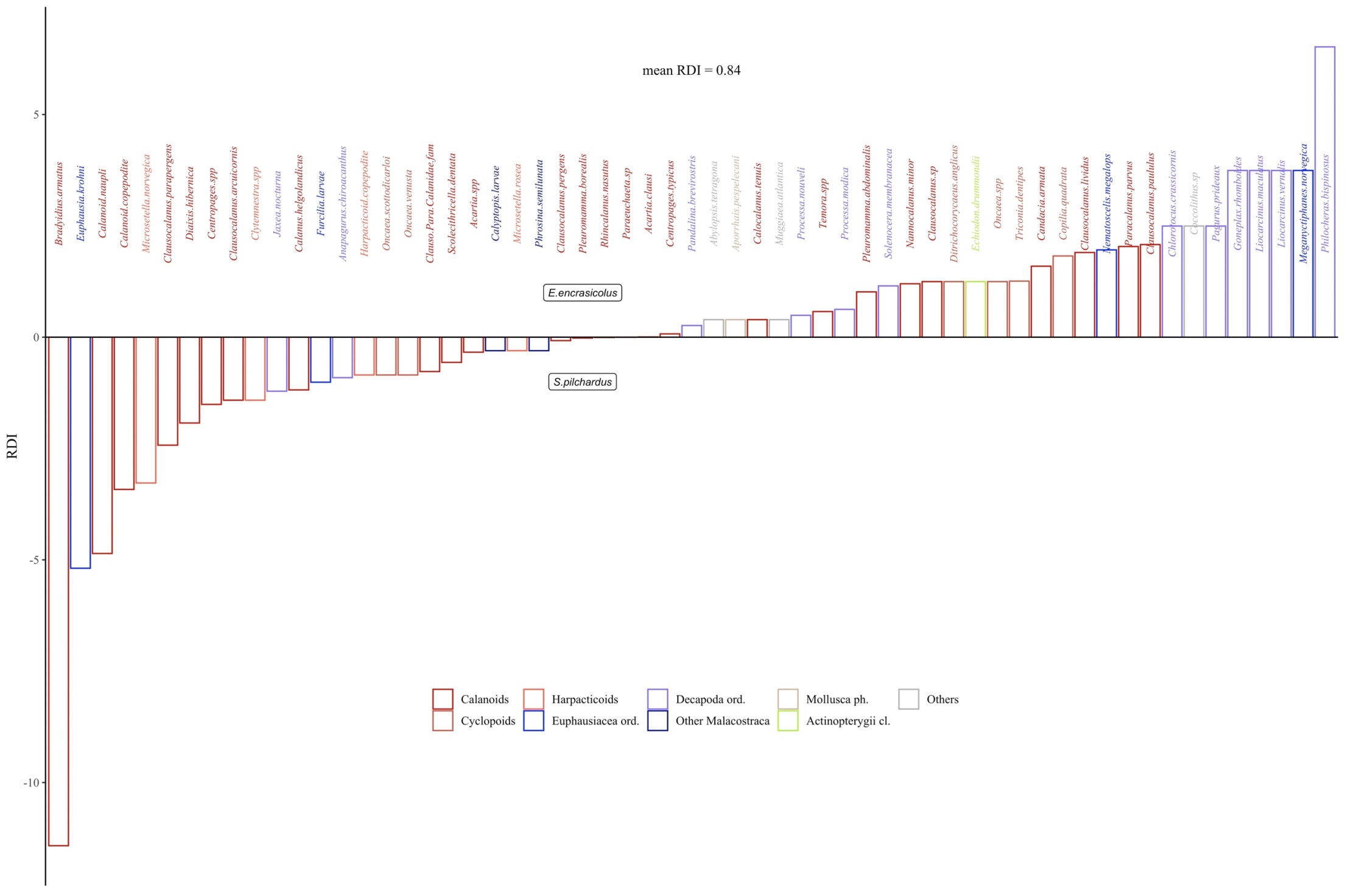
**

**Figure S3.** Paired comparisons of the Specific Relative Dissimilarity Index (RDI) between predator fish species, considering corrected diet characterization. Prey groups with high RDI are those that make the diet of the two predator species more different; in contrast, prey groups at the centre of graphs (with low RDI values) are those for which diet overlap occurs. RDI values in the top of each plot represent the mean Dissimilarity Index for each paired comparison.


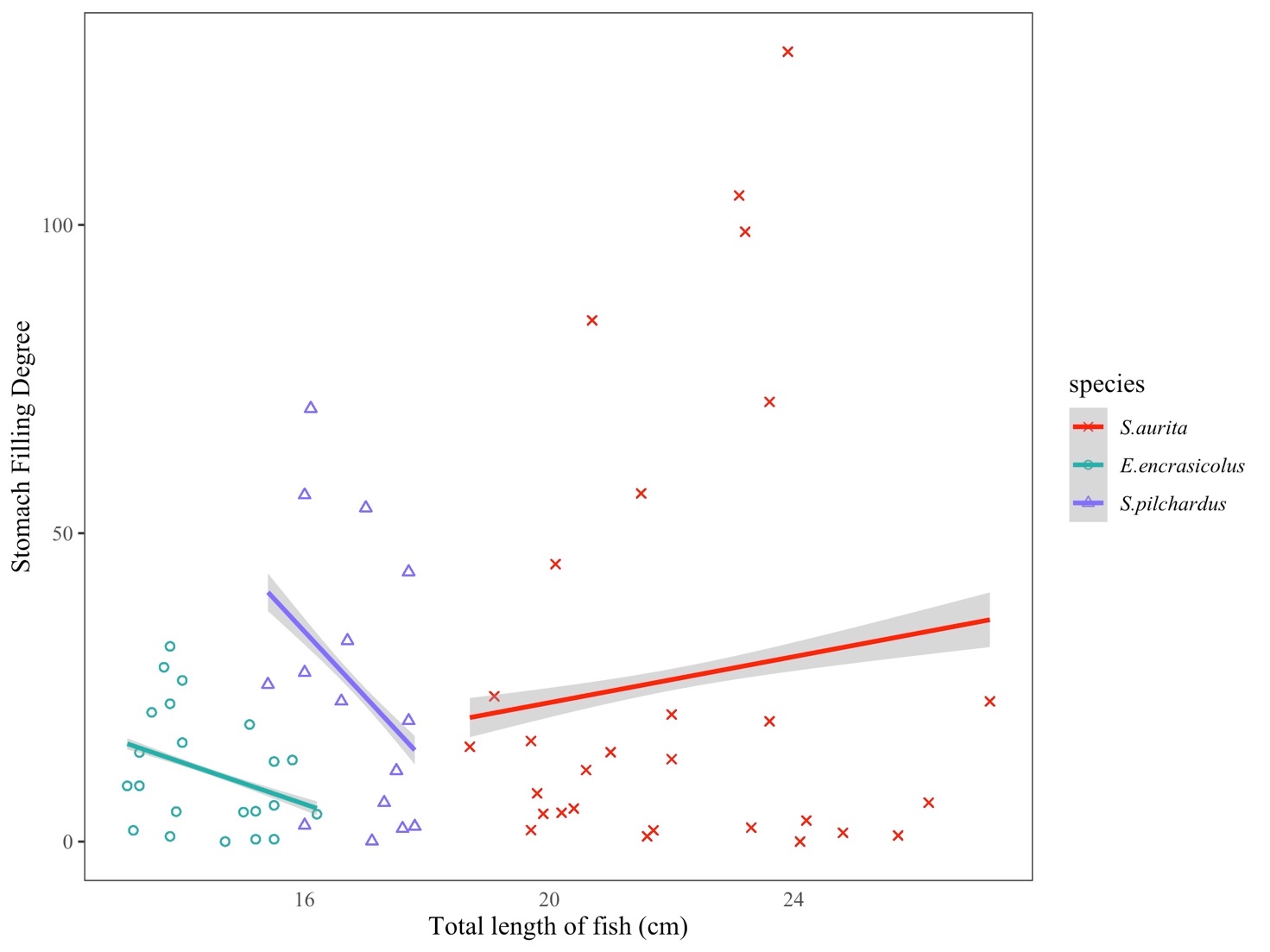


**Figure S4.** Linear regressions and confidence intervals (in shaded grey) for the stomach filling degree compared to the total length of fish for the three species. Each dot represents one fish.


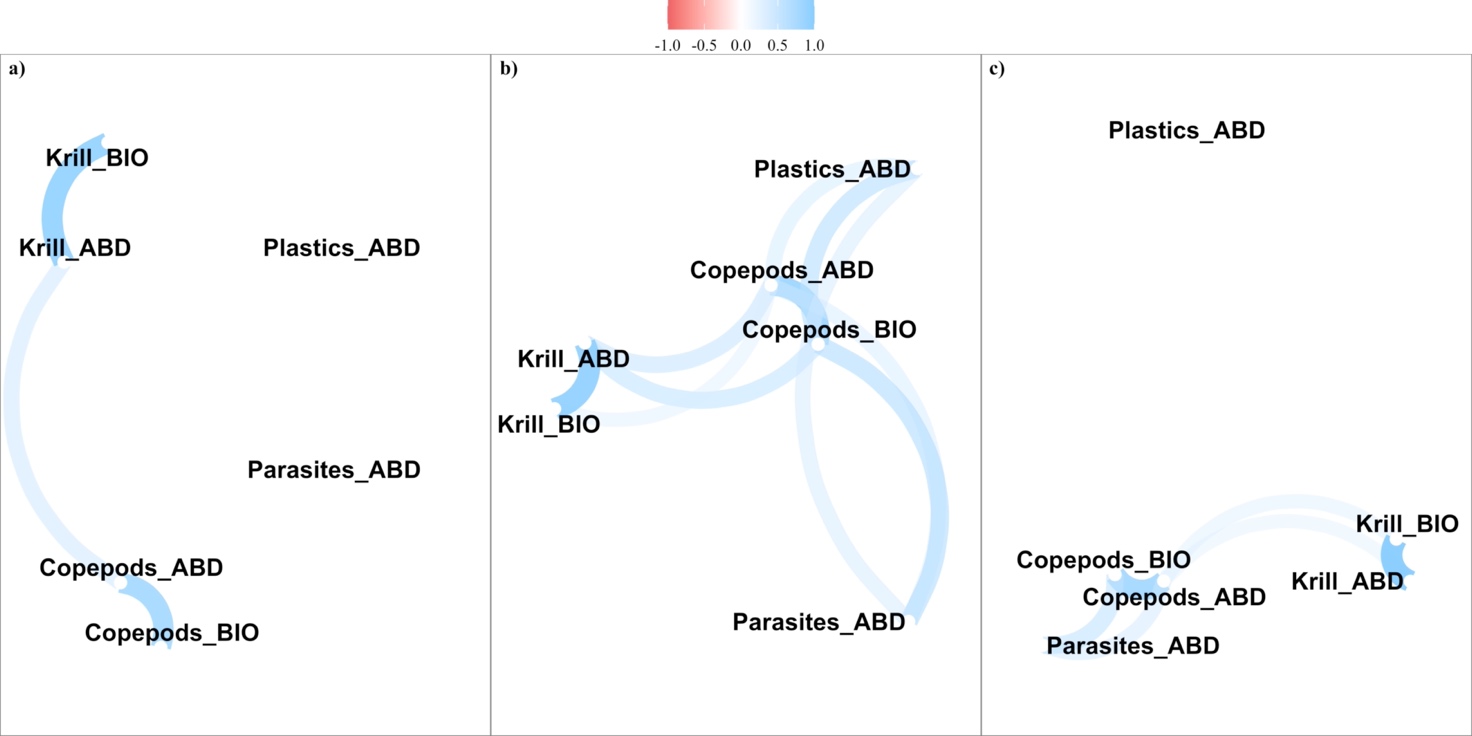


**Figure S5.** Network Spearman correlation plots for **(a)** sardinella (*S. aurita*), **(b)** anchovy (*E. encrasicolus*) and **(c)** sardine (*S. pilchardus*). In these plots variables that are more highly correlated appear closer together and are joined by stronger paths. Paths are also colored by their sign (blue for positive and red for negative correlation). Only significant (α = 0.05) Spearman correlation are showed in the network plots.


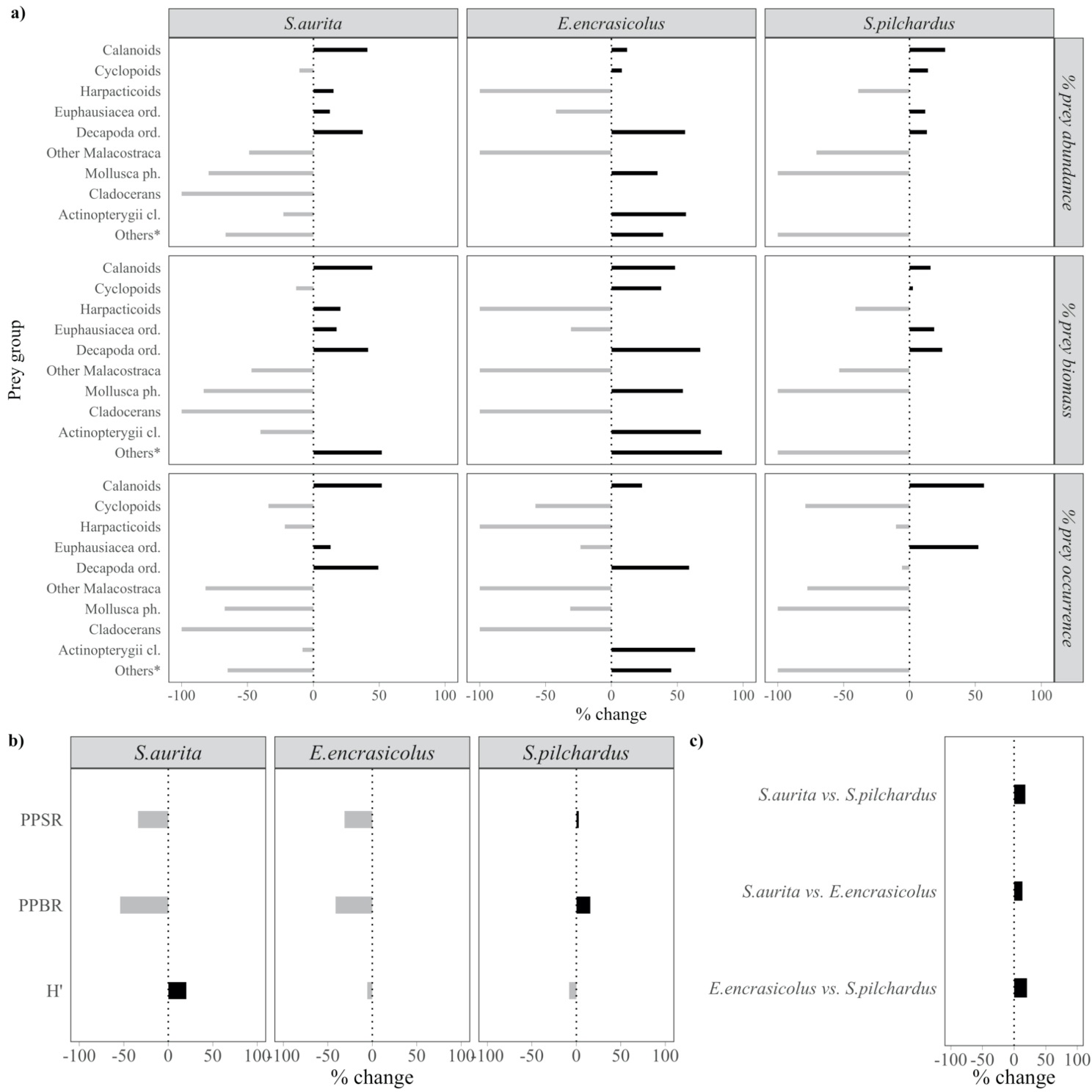


**Figure S6.** Sensitivity analysis for different parameters estimated from microscope diet characterization in comparison with the corrected diet characterization applied in the study: **(a)** Diet characterization based on merged prey groups, **(b)** mean PPSR, PPBR and H’ values per fish species (see detailed sensitivity analysis of PPSR and PPBR in Fig. S6), and **(c)** mean diet dissimilarity values in paired comparison between fish species. Positive (in black) and negative (in grey) %change values mean higher and lower values for corrected characterization compared with microscope characterization, respectively. * ‘Others’ group refers to the corresponding prey group based on microscope analysis, since the one for corrected characterization has been broken down and presented as a separated analysis (Fig. 3a).


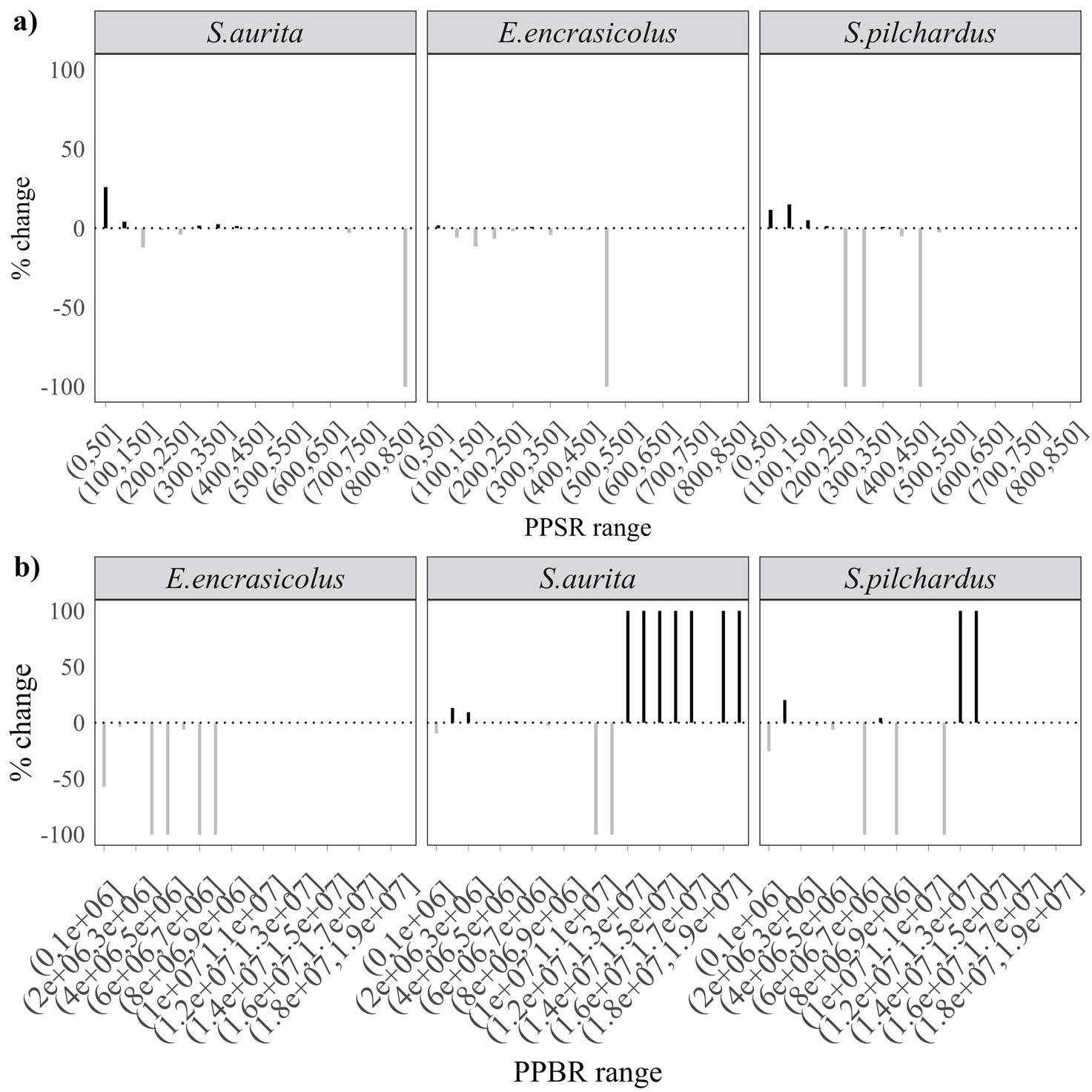


**Figure S7.** Sensitivity analysis for **(a)** predator-prey size ratio (PPSR) and **(b)** predator-prey biomass ratio (BBBR) estimated from microscope diet characterization in comparison with the corrected diet characterization applied in the study. Positive (in black) and negative (in grey) % change values mean higher and lower values for corrected diet characterization compared to microscope characterization, respectively.


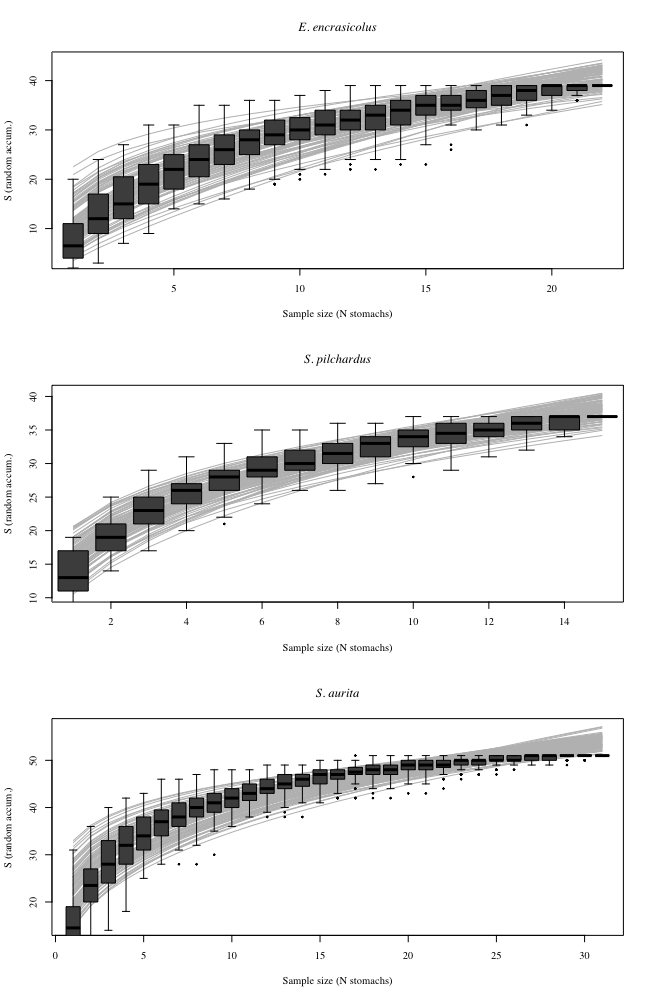


**Figure S8.** Rarefaction curves of the stomach content sampling, showing the coverage of the total prey richness (S, as random accumulations) with the actual sample size (N stomachs) in the three species.

**Table S1.** Parameters obtained from t-tests comparing sampling locations, to test for changes of δ^15^N and δ^13^C in round sardinella.

| ***SIA variable*** |  | ***Df*** | ***Sum Sq*** | ***Mean Sq*** | ***F value*** | ***P*** |
| --- | --- | --- | --- | --- | --- | --- |
| *δ^15^N* | Stations | 1 | 0.281 | 0.2813 | 1.206 | **0.289** |
|  | Residuals | 15 | 3.499 | 0.2333 |  |  |
|  |  |  |  |  |  |  |
| *δ13C* | Stations | 1 | 0.0239 | 0.02385 | 0.259 | **0.618** |
|  | Residuals | 15 | 1.38 | 0.092 |  |  |

**Table S2.** Definition of prey groups identified in stomach contents under the microscope, with DNA metabarcoding, the assigned wet weight (see Methods), and (merged) classification of groups for graphical presentation. ^A^ denotes taxa identified in both microscope and DNA metabarcoding; ^B^ denotes taxa determined under the microscope and assigned to species level based on data from DNA metabarcoding; ^C^ denotes taxa determined under the microscope but not detected with DNA metabarcoding; ^D^ denotes taxa detected with DNA metabarcoding and not determined under the microscope. ‘CORR’ means that correction procedure was applied to the undetermined group in microscope analysis, for which proportionally re-assigned prorated values were considered according to the detected species with DNA metabarcoding (see Methods). ‘*’ indicates assigned species after wrong or uncertain identification under the microscope.

| ***Merged group*** | ***Prey species/group (microscope)*** | ***Prey species/group***  ***(DNA metabarcoding)*** | ***Wet weight (mg)*** |
| --- | --- | --- | --- |
| Calanoids | Calanoid naupli^C^ | - | 0.004 |
|  | Calanoid copepodite^B,C^ | CORR [*Bradyidius armatus, Diaixis hibernica, Lucicutia flavicornis, Nannocalanus minor, Paracalanus parvus, Pleuromamma borealis, Rhincalanus nasutus*]^B^ | 0.036 |
|  | Unidentified Calanoid^B,C^ | CORR [*Acartia clausi, Bradyidius armatus, Calanus euxinus, Calanus helgolandicus, Calocalanus contractus, Calocalanus styliremis, Clausocalanus arcuicornis, Clausocalanus lividus, Clausocalanus parapergens, Clausocalanus paululus, Clausocalanus pergens, Diaixis hibernica, Lucicutia flavicornis, Mecynocera clausi, Mesocalanus tenuicornis, Nannocalanus minor, Paracalanus parvus, Paraeuchaeta sp., Pleuromamma borealis, Pleuromamma gracilis, Rhincalanus nasutus, Subeucalanus pileatus, Temora stylifera*]^B^ | 0.224 |
|  | *Acartia* spp.^B,C^ | *Acartia clausi*^B^*, Mecynocera clausi*^B^ | 0.019 |
|  | *Calanus gracilis*^B,C^ | *Pleuromamma gracilis*^B^ | 0.318 |
|  | *Calanus helgolandicus*^A,C^ | *Calanus helgolandicus*^A^ | 0.318 |
|  | *Calocalanus contractus*^A,B^ | *Calocalanus contractus*^A,B^*, Calocalanus styliremis*^B^ | 0.857 |
|  | *Candacia armata*^A,B,C^ | *Bradyidius armatus*^B^*, Candacia armata*^A^*, Scolecithricella dentata*^B^ | 0.773 |
|  | *Centropages* spp.^B,C^ | *Centropages typicus*^B^ | 0.219 |
|  | *Centropages typicus*^A,C^ | *Centropages typicus*^A^ | 0.219 |
|  | *Centropages chierchiae*^B,C^ | *Centropages typicus*^B^ | 0.219 |
|  | Clauso-/Para-/Calanidae fam.^B,C^ | CORR [*Clausocalanus arcuicornis, Clausocalanus lividus, Clausocalanus pergens*]^B^ | 0.318 |
|  | *Clausocalanus* spp.^B,C^ | *Clausocalanus arcuicornis*^B^*, Clausocalanus lividus*^B^*, Clausocalanus paululus*^B^*, Clausocalanus pergens*^B^ | 0.736 |
|  | *Paraeuchaeta* spp.^A^ | *Paraeuchaeta* spp.^A^ | 0.996 |
|  | *Pleuromamma borealis*^A,C^ | *Pleuromamma borealis*^A,4^*, Pleuromamma gracilis*^B,D^ | 0.318 |
|  | *Temora* spp.^B,C^ | *Temora stylifera*^B^ | 0.246 |
|  | *Temora stylifera*^A,C^ | *Temora stylifera*^A^ | 0.246 |
|  |  |  |  |
| Cyclopoids | Cyclopoid naupli^C^ | - | 0.004 |
|  | Cyclopoid copepodite^B,C^ | *Copilia quadrata*^B^ | 0.032 |
|  | *Ditrichocorycaeus anglicus*^A,C^ | *Ditrichocorycaeus anglicus*^A^ | 0.072 |
|  | *Oncaea* spp.^B,C^ | *Oncaea mediterranea*^B^*, O. scottodicarloi*^B^*, Oncaea venusta*^B^ | 0.016 |
|  | *-* | *Triconia dentipes*^D^ | - |
|  |  |  |  |
| Harpacticoids | Harpacticoid copepodite^C^ | - | 0.034 |
|  | *Clytemnestra spp.*^C^ | - |  |
|  | *Euterpina acutifrons*^C^ | - | 0.031 |
|  | *Microsetella rosea*^C^ | *Microsetella* spp.^B^ | 0.137 |
|  | *Microsetella norvegica*^A,C^ | *Microsetella norvegica*^A,D^ | 0.137 |
|  |  |  |  |
| Euphausiacea ord. | Metanauplius^C^ | - | 0.005 |
|  | Calyptopis larvae^B,C^ | CORR [*Eualus cranchii, Euphausia krohni, Galathea sp., Nematoscelis megalops, Philocheras bispinosus, Processa modica, Processa nouveli, Solenocera membranacea*]^B^ | 5.621 |
|  | Furcilia larvae^B,C^ | CORR [*Euphausia krohni, Galathea sp., Nematoscelis megalops, Pasiphaea sivado, Philocheras bispinosus, Processa nouveli*]^B^ | 5.621 |
|  | Unidentified Euphausiid^B,C^ | CORR [*Euphausia krohni, , Goneplax rhomboides*, Meganyctiphanes norvegica, Nematoscelis megalops*, Philocheras bispinosus*, Processa modica*, Processa nouveli**]^B^ | 11.795 |
|  | *Euphausia krohni*^A,B,C^ | *Euphausia krohni*^A^*,*  CORR [*Meganyctiphanes norvegica, , Nematoscelis megalops, Philocheras bispinosus*, Processa modica*, Processa nouveli**]^B^ | 11.795 |
|  | *Meganyctiphanes norvegica*^A,B,C^ | *Meganyctiphanes norvegica*^A^*,*  CORR [*Euphausia krohni, Goneplax rhomboides*, Nematoscelis megalops, Processa modica*, Processa nouveli**]^B^ | 11.795 |
|  |  |  |  |
| Decapoda ord. | Decapod Zoea larvae^B,C^ | CORR [*Galathea sp., Pandalina brevirostris, Philocheras bispinosus, Processa nouveli, Solenocera membranacea*]^B^ | 3.284 |
|  | Decapod Megalopa larvae^B,C^ | CORR [*Galathea sp., Liocarcinus maculatus, Philocheras bispinosus, Processa nouveli*]^B^ | 3.284 |
|  | Decapod Late Larvae^B,C^ | CORR [*Anapagurus breviaculeatus, Anapagurus chiroacanthus, Chlorotocus crassicornis, Eualus cranchii, Galathea sp., Goneplax rhomboides, Inachus dorsettensis, Jaxea nocturna, Liocarcinus vernalis, Nematoscelis megalops*, Pagurus forbesii, Pagurus prideaux, Pandalina brevirostris, Philocheras bispinosus, Processa modica, Processa nouveli, Solenocera membranacea*]^B^ | 5.621 |
|  | Unidentified Decapod^C^ | CORR [*Anapagurus chiroacanthus, Galathea sp., Inachus dorsettensis, Jaxea nocturna, Liocarcinus depurator, Liocarcinus vernalis, Liocarcinus zariquieyi, Pandalina brevirostris, Philocheras bispinosus, Processa nouveli, Solenocera membranacea*]^B^ | 8.747 |
|  | - | *Anapagurus laevis*^D^*, Ebalia cranchii*^D^*, Euphysa aurata*^D^, *Eurynome spinosa*^D^*, Eusergestes arcticus*^D^ | - |
|  |  |  |  |
| Other Malacostraca | Unidentified Amphipod^C^ | CORR [*Philocheras bispinosus, Processa nouveli, Pandalina brevirostris*]^B^ | 18.935 |
|  | Unidentified Malacostraca^B,C^ | CORR [*Anapagurus breviaculeatus, Anapagurus chiroacanthus, Eualus cranchii, Euphausia krohni*, Eurynome spinosa, Galathea sp., Inachus dorsettensis, Jaxea nocturna, Liocarcinus depurator, Nematoscelis megalops*, Pandalina brevirostris, Philocheras bispinosus, Phrosina semilunata, Processa nouveli, Solenocera membranacea, Vibilia armata*]^B^ | 11.795 |
|  | Unidentified Mysid^C^ | - | 11.795 |
|  |  |  |  |
| Crustacean remains | Cirripedia naupli^B^ | *Copilia quadrata*^B,D^ | 0.028 |
|  | Cirripedia^C^ | - | 0.029 |
|  | - | *Archiconchoecia striata*^D^*, Porroecia spinirostris*^D^ | - |
|  |  |  |  |
| Mollusca ph. | Bivalve veliger^B,C^ | *Abra nitida*^B,D^*, Spisula subtruncata*^B^ | 0.012 |
|  |  | *Corbula gibba*^D^ | - |
|  | Gastropod veliger^B,C^ | *Aporrhais pespelecani*^B^ | 0.031 |
|  |  | *Cavolinia inflexa*^D^*, Euspira nitida*^D^ | - |
|  |  |  |  |
| Cladocerans | *Penilia avirostris*^C^ | *-* | 0.087 |
|  | *Podon* spp.^C^ | *-* | 0.087 |
|  |  | *Podon intermedius*^D^ | - |
|  |  |  |  |
| Actinopterygii cl. | Fish egg: Unidentified fish^B,C^ | *Benthosema glaciale*^B,D^*, Spicara maena*^B^ | 3.066 |
|  | Fish larvae: Unidentified fish^B,C^ | *Echiodon.drummondii*^B^ | 7.648 |
|  | - | *Merluccius merluccius*^D^*, Mullus barbatus*^D^*, Trisopterus capelanus*^D^ | - |
|  |  |  |  |
| Others | Crustacean egg^C^ | *-* | *-* |
|  | Foraminifera ord.^C^ | *-* | - |
|  | Siphonophorae ord.^B^ | *Abylopsis tetragona*^B,D^*, Leuckartiara octona*^B,D^*, Muggiaea atlantica*^B,D^ | 4.014 |
|  |  |  |  |
| [Others]^D^ | - | Annelida ph.: *Magelona* sp.*, Owenia fusiformis, Phyllodoce rosea, Spiochaetopterus costarum*  Chaetognatha ph.: *Pseudosagitta lyra*  Cnidaria ph. - Hydrozoa cl.  Actiniaria ord. (Anthozoa): *Abylopsis tetragona*, *Aglaura hemistoma, Bougainvilla muscus, Clytia hemisphaerica, Corymorpha* sp.*, Corymorpha sarsii, Eutima gegenbauri, Helgicirrha cari, Leuckartiara octona, Lizzia blondina, Nanomia bijunga, Obelia dichotoma, Obelia geniculata, Podocoryna aerolata*  Siphonophorae ord.: *Abylopsis tetragona, Muggiaea atlantica, Sphaeronectes koellikeri, Sphaeronectes irregularis*  Echinodermata ph.: *Gracilechinus acutus, Holothuria forskali, Luidia sarsi, Paracentrotus lividus*  Haptophyta ph.: *Coccolithus* sp.  Mollusca ph.: *Cavolinia inflexa*  Nemertea ph.: Tubulanidae fam.  Phoronida ph.: *Phoronis muelleri*  Porifera ph. | - |
|  |  |  |  |

**Table S3.** **(a)** Standard Ellipses Areas corrected for small samples size (SEA_C_) and Bayesian Standard Ellipses Areas (SEA_B_). **(b)** Bayesian Standard Ellipses Areas Overlap. Credible intervals are given in brackets.

| **(a)** |  |  |  |  |
| --- | --- | --- | --- | --- |
|  | **Species** | **SEA_C_** | **SEA_B_** |  |
|  | *S. aurita* | 0.48 | 0.46 [0.39-0.55] |  |
|  | *E. encrasicolus* | 0.22 | 0.21 [0.18-0.25] |  |
|  | *S. pilchardus* | 0.40 | 0.38 [0.32-0.46] |  |
| **(b)** |  |  |  |  |
|  |  | *S. aurita* | *E. encrasicolus* | *S. pilchardus* |
|  | *S. aurita* |  | 21.00 [0-47.94] | 20.30 [0-49.97] |
|  | *E. encrasicolus* | 44.81 [0-94.91] |  | 17.99 [0-71.68] |
|  | *S. pilchardus* | 26.37 [0-61.51] | 10.98 [0-41.44] |  |

**Table S4.** Diet composition of sardinella (*S. aurita*), anchovy (*E. encrasicolus*) and sardine (*S. pilchardus*), presented as means of numerical prey frequency (%N), estimated biomass (%B) and frequency of occurrence (%O), by method (*micr*: microscope analysis; *CORR*: corrected values from the combination of microscope and DNA metabarcoding data [see Methods] and *DNA*: DNA metabarcoding). ‘-’ means no item (0.00%). Number of samples (N) for each category is presented in Table 1.

|  |  | ***S. aurita*** | | | | | | |  | ***E. encrasicolus*** | | | | | | |  | ***S. pilchardus*** | | | | | | |
| --- | --- | --- | --- | --- | --- | --- | --- | --- | --- | --- | --- | --- | --- | --- | --- | --- | --- | --- | --- | --- | --- | --- | --- | --- |
|  |  | **%N** | | **%B** | | **%O** | | |  | **%N** | | **%B** | | **%O** | | |  | **%N** | | **%B** | | **%O** | | |
| **Prey group (species)⇓ method⇒** |  | ***micr*** | ***CORR*** | ***micr*** | ***CORR*** | ***micr*** | ***CORR*** | ***DNA*** |  | ***micr*** | ***CORR*** | ***micr*** | ***CORR*** | ***micr*** | ***CORR*** | ***DNA*** |  | ***micr*** | ***CORR*** | ***micr*** | ***CORR*** | ***micr*** | ***CORR*** | ***DNA*** |
| *Abra nitida* |  | - | 0.05 | - | <0.01 | - | 0.27 | 0.42 |  | - | - | - | - | - | - | - |  | - | - | - | - | - | - | - |
| *Abylopsis tetragona* |  | - | 0.29 | - | 0.49 | - | 0.27 | 2.24 |  | - | 0.77 | - | 1.52 | - | 1.41 | 2.50 |  | - | - | - | - | - | - | - |
| *Acartia clausi* |  | - | 0.49 | - | <0.01 | - | 2.75 | 2.38 |  | - | 0.39 | - | <0.01 | - | 1.41 | 1.88 |  | - | 0.83 | - | <0.01 | - | 2.38 | 2.03 |
| *Acartia* spp. |  | 0.59 | 0.24 | <0.01 | <0.01 | 3.00 | 0.55 | 2.24 |  | 1.84 | 2.70 | <0.01 | 0.01 | 3.73 | 4.23 | - |  | 2.58 | 3.00 | 0.01 | 0.01 | 4.21 | 4.76 | - |
| *Aglaura hemistoma* |  | - | - | - | - | - | - | 0.84 |  | - | - | - | - | - | - | 1.25 |  | - | - | - | - | - | - | - |
| *Anapagurus breviaculeatus* |  | - | 0.10 | - | 0.11 | - | 0.27 | 0.14 |  | - | - | - | - | - | - | - |  | - | 5.16 | - | 9.28 | - | 1.19 | 0.68 |
| *Anapagurus chiroacanthus* |  | - | - | - | - | - | - | - |  | - | - | - | - | - | - | - |  | - | - | - | - | - | - | 0.68 |
| *Anapagurus laevis* |  | - | - | - | - | - | - | 0.14 |  | - | - | - | - | - | - | - |  | - | - | - | - | - | - | - |
| *Aporrhais pespelecani* |  | - | - | - | - | - | - | - |  | - | 3.09 | - | 0.02 | - | 1.41 | 0.63 |  | - | - | - | - | - | - | - |
| *Archiconchoecia striata* |  | - | - | - | - | - | - | 0.14 |  | - | - | - | - | - | - | - |  | - | - | - | - | - | - | - |
| *Benthosema glaciale* |  | - | 0.05 | - | 0.03 | - | 0.27 | 0.42 |  | - | - | - | - | - | - | - |  | - | - | - | - | - | - | - |
| Bivalve veliger |  | 0.48 | - | <0.01 | - | 2.77 | - | - |  | 0.17 | - | <0.01 | - | 0.62 | - | - |  | 0.16 | - | <0.01 | - | 1.05 | - | - |
| *Bougainvillia muscus* |  | - | - | - | - | - | - | 0.98 |  | - | - | - | - | - | - | 1.25 |  | - | - | - | - | - | - | 6.76 |
| *Bradyidius armatus* |  | - | 0.24 | - | 0.05 | - | 0.82 | 0.70 |  | - | - | - | - | - | - | 1.88 |  | - | 2.83 | - | 0.27 | - | 9.52 | 6.08 |
| Calanoid copepodite |  | 1.85 | 1.02 | <0.01 | <0.01 | 4.85 | 2.20 | 1.68 |  | 3.01 | - | <0.01 | - | 5.59 | - | 0.63 |  | 5.07 | 1.66 | 0.01 | <0.01 | 7.37 | 3.57 | 0.68 |
| Calanoid naupli |  | 0.36 | 0.63 | <0.01 | <0.01 | 1.85 | 2.20 | - |  | 0.33 | - | <0.01 | - | 0.62 | - | - |  | 2.42 | 4.33 | <0.01 | <0.01 | 4.21 | 7.14 | - |
| *Calanus euxinus* |  | - | 0.05 | - | <0.01 | - | 1.10 | 0.98 |  | - | - | - | - | - | - | - |  | - | - | - | - | - | - | - |
| *Calanus gracilis* |  | 0.08 | 0.15 | <0.01 | <0.01 | 0.46 | 0.55 | <0.01 |  | 5.18 | - | <0.01 | - | 5.59 | - | - |  | - | - | - | - | - | - | - |
| *Calanus helgolandicus* |  | 1.48 | 2.62 | 0.09 | 0.16 | 3.93 | 4.67 | 1.96 |  | 0.84 | 2.32 | 0.28 | 0.15 | 0.62 | 4.23 | 2.50 |  | 1.85 | 1.66 | 0.12 | 0.11 | 3.68 | 5.95 | 1.35 |
| *Calocalanus contractus* |  | 0.17 | 1.02 | 0.03 | 0.06 | 0.69 | 3.30 | 1.82 |  | 0.33 | 1.93 | 0.12 | 0.38 | 1.24 | 1.41 | - |  | - |  | - | - | - | - | - |
| *Calocalanus styliremis* |  | - | 0.19 | - | 0.03 | - | 0.82 | 0.98 |  | - | - | - | - | - | - | - |  | - | - | - | - | - | - | - |
| Calyptopis larvae |  | 30.10 | - | 30.72 | - | 5.08 | - | - |  | 5.02 | - | 0.32 | - | 3.11 | - | - |  | 13.77 | 0.17 | 15.36 | 0.19 | 5.79 | 1.19 | - |
| *Candacia armata* |  | 2.10 | 2.62 | 0.38 | 0.50 | 3.70 | 2.75 | 1.12 |  | 5.18 | 10.42 | 0.84 | 2.36 | 5.59 | 4.23 | - |  | 1.61 | - | 0.32 | - | 3.68 | - | - |
| *Cavolinia inflexa* |  | - | - | - | - | - | - | - |  | - | - | - | - | - | - | 0.63 |  | - | - | - | - | - | - | - |
| *Centropages chierchiae* |  | 0.14 | 0.19 | <0.01 | <0.01 | 1.15 | 1.10 | <0.01 |  | - | - | - | - | - | - | - |  | - | - | - | - | - | - | - |
| *Centropages* spp. |  | 0.34 | 0.34 | <0.01 | <0.01 | 1.62 | 1.10 | <0.01 |  | 0.17 | - | <0.01 | - | 0.62 | - | - |  | 0.24 | 0.50 | <0.01 | 0.01 | 1.05 | 2.38 | - |
| *Centropages typicus* |  | 0.78 | 1.31 | 0.02 | 0.03 | 2.31 | 1.65 | 0.14 |  | 0.33 | 0.39 | <0.01 | 0.01 | 1.24 | 1.41 | - |  | 0.08 | 0.17 | <0.01 | <0.01 | 0.53 | 1.19 | - |
| *Chlorotocus crassicornis* |  | - | - | - | - | - | - | - |  | - | 0.77 | - | 0.99 | - | 1.41 | 0.63 |  | - | - | - | - | - | - | - |
| Cirripedia |  | 0.08 | - | <0.01 | - | 0.46 | - | - |  | - | - | - | - | - | - | - |  | 0.08 | - | <0.01 | - | 0.53 | - | - |
| Cirripedia naupli |  | 0.06 | - | <0.01 | - | 0.23 | - | - |  | - | - | - | - | - | - | - |  | - | - | - | - | - | - | - |
| Clauso-/Para-calanidae fam. |  | 1.15 | 0.92 | 0.07 | 0.06 | 3.93 | 1.92 | - |  | 0.33 | - | 0.02 | - | 1.24 | - | - |  | 1.29 | 0.67 | 0.08 | 0.04 | 3.68 | 1.19 | - |
| *Clausocalanus arcuicornis* |  | - | 0.10 | - | <0.01 | - | 0.82 | 1.40 |  | - | - | - | - | - | - | - |  | - | 0.17 | - | <0.01 | - | 1.19 | 0.68 |
| *Clausocalanus lividus* |  | - | 0.49 | - | 0.03 | - | 3.02 | 2.66 |  | - | 2.32 | - | 0.31 | - | 4.23 | 2.50 |  | - | 0.33 | - | <0.01 | - | 1.19 | 1.35 |
| *Clausocalanus parapergens* |  | - | - | - | - | - | - | - |  | - | - | - | - | - | - | - |  | - | 1.33 | - | 0.05 | - | 1.19 | 1.35 |
| *Clausocalanus paululus* |  | - | <0.01 | - | <0.01 | - | 0.82 | 0.84 |  | - | 0.39 | - | 0.06 | - | 2.82 | 1.88 |  | - | - | - | - | - | - | 2.70 |
| *Clausocalanus pergens* |  | - | 1.07 | - | 0.07 | - | 3.85 | 2.66 |  | - | 1.16 | - | 0.06 | - | 5.63 | 3.75 |  | - | 3.16 | - | 0.20 | - | 4.76 | 5.41 |
| *Clausocalanus* sp. |  | 0.14 | - | 0.02 | - | 0.69 | - | - |  | 2.01 | 0.39 | 0.25 | 0.06 | 4.35 | 1.41 | - |  | 0.40 | - | 0.05 | - | 2.11 | - | - |
| *Clytia hemisphaerica* |  | - | - | - | - | - | - | 3.09 |  | - | - | - | - | - | - | 2.50 |  | - | - | - | - | - | - | - |
| *Coccolithus* sp. |  | - | 0.15 | - | <0.01 | - | 0.27 | 1.40 |  | - | 0.39 | - | <0.01 | - | 1.41 | 3.13 |  | - | - | - | - | - | - | 0.68 |
| *Copilia quadrata* |  | - | 0.73 | - | <0.01 | - | 0.82 | 2.38 |  | - | 1.54 | - | <0.01 | - | 2.82 | 6.25 |  | - | - | - | - | - | - | 3.38 |
| *Corbula gibba* |  | - | - | - | - | - | - | - |  | - | - | - | - | - | - | - |  | - | - | - | - | - | - | 0.68 |
| *Corymorpha sarsii* |  | - | - | - | - | - | - | 1.40 |  | - | - | - | - | - | - | - |  | - | - | - | - | - | - | - |
| *Corymorpha* sp. |  | - | - | - | - | - | - | 0.84 |  | - | - | - | - | - | - | 1.88 |  | - | - | - | - | - | - | 2.70 |
| Cyclopoid copepodite |  | 0.50 | - | <0.01 | - | 0.92 | - | - |  | 0.33 | - | <0.01 | - | 0.62 | - | - |  |  | - |  | - |  | - | - |
| Cyclopoid.naupli |  | - | - | - | - | - | - | - |  | - | - | - | - | - | - | - |  | 0.08 | - | <0.01 | - | 0.53 | - | - |
| Decapod late larvae |  | 3.27 | - | 3.34 | - | 5.31 | - | - |  | 6.35 | - | 6.00 | - | 5.59 | - | - |  | 3.46 | - | 3.86 | - | 5.26 | - | - |
| Decapod megalopa larvae |  | 0.11 | 0.05 | 0.07 | 0.03 | 0.92 | 0.27 | - |  | 1.51 | - | 0.83 | - | 1.24 | - | - |  | - | - | - | - | - | - | - |
| Decapod zoea larvae |  | 0.98 | 0.92 | 0.58 | 0.58 | 3.23 | 2.20 | - |  | 0.84 | - | 0.46 | - | 1.86 | - | - |  | - | - | - | - | - | - | - |
| *Diaixis hibernica* |  | - | 2.96 | - | 0.12 | - | 6.04 | 3.93 |  | - | 2.32 | - | 0.12 | - | 7.04 | 5.00 |  | - | 8.15 | - | 0.23 | - | 11.90 | 6.76 |
| *Ditrichocorycaeus anglicus* |  | 0.53 | 0.24 | <0.01 | <0.01 | 3.00 | 0.82 | 0.28 |  | 0.17 | 0.39 | <0.01 | <0.01 | 0.62 | 1.41 | 1.25 |  | 0.97 | - | 0.01 | - | 3.16 | - | 3.38 |
| *Ebalia cranchii* |  | - | - | - | - | - | - | - |  | - | - | - | - | - | - | - |  | - | - | - | - | - | - | 0.68 |
| *Echiodon drummondii* |  | - | - | - | - | - | - | - |  | - | 0.39 | - | 0.67 | - | 1.41 | 0.63 |  | - | - | - | - | - | - | - |
| Egg: undefined crustacean |  | 1.85 | - | <0.01 | - | 1.39 | - | - |  | 0.84 | - | <0.01 | - | 1.24 | - | - |  | - | - | - | - | - | - | - |
| *Eualus cranchii* |  | - | 3.69 | - | 4.01 | - | 1.10 | 0.70 |  | - | - | - | - | - | - | - |  | - | - | - | - | - | - | - |
| *Euphausia krohni* |  | 9.12 | 19.48 | 19.53 | 42.54 | 1.62 | 1.37 | 1.68 |  | 9.87 | 3.47 | 19.56 | 9.30 | 6.83 | 2.82 | 1.25 |  | 2.09 | 29.45 | 4.90 | 70.52 | 1.58 | 8.33 | 5.41 |
| *Euphysa aurata* |  |  |  |  |  |  |  | 0.28 |  | - | - | - | - | - | - | - |  | - | - | - | - | - | - | 1.35 |
| *Eurynome spinosa* |  | - | - | - | - | - | - | - |  | - | - | - | - | - | - | 0.63 |  | - | - | - | - | - | - | 0.68 |
| *Eusergestes arcticus* |  |  |  |  |  |  |  | 0.14 |  | - | - | - | - | - | - | 0.63 |  | - | - | - | - | - | - | - |
| *Euspira nitida* |  | - | - | - | - | - | - | - |  | - | - | - | - | - | - | 0.63 |  | - | - | - | - | - | - | - |
| *Euterpina acutifrons* |  | 0.06 | 0.05 | <0.01 | <0.01 | 0.46 | 0.27 | - |  | - | - | - | - | - | - | - |  | 0.08 | - | <0.01 | - | 0.53 | - | - |
| *Eutima gegenbauri* |  | - | - | - | - | - | - | 0.14 |  | - | - | - | - | - | - | - |  | - | - | - | - | - | - | - |
| Fish egg (unidentified) |  | 0.20 | - | 0.09 | - | 1.62 | - | - |  | - | - | - | - | - | - | - |  | - | - | - | - | - | - | - |
| Fish larvae (unidentified) |  | 0.06 | - | 0.08 | - | 0.46 | - | - |  | 0.17 | - | 0.21 | - | 0.62 | - | - |  | - | - | - | - | - | - | - |
| Foraminifera ord. |  | 0.39 | - | <0.01 | - | 0.92 | - | - |  | - | - | - | - | - | - | - |  | 0.08 | - | <0.01 | - | 0.53 | - | - |
| Furcilia larvae |  | 1.26 | - | 0.26 | - | 2.77 | - | - |  | 3.51 | - | 0.67 | - | 2.48 | - | - |  | 1.77 | 0.33 | 0.40 | 0.08 | 3.68 | 2.38 | - |
| *Galathea* sp. |  | - | 5.83 | - | 6.52 | - | 3.30 | 2.66 |  | - | - | - | - | - | - | - |  | - | - | - | - | - | - | - |
| Gastropod veliger |  | 0.48 | 0.05 | <0.01 | <0.01 | 1.85 | 0.27 | - |  | 1.84 | - | <0.01 | - | 2.48 | - | - |  | 0.40 | - | <0.01 | - | 2.11 | - | - |
| *Goneplax rhomboides* |  | - | 0.10 | - | 0.11 | - | 0.55 | 0.42 |  | - | 0.39 | - | 1.03 | - | 1.41 | 0.63 |  | - | - | - | - | - | - | - |
| *Gracilechinus acutus* |  | - | - | - | - | - | - | - |  | - | - | - | - | - | - | 0.63 |  | - | - | - | - | - | - | - |
| Harpacticoid copepodite |  | - | - | - | - | - | - | - |  | 0.17 | - | <0.01 | - | 0.62 | - | - |  | 0.56 | 0.67 | <0.01 | <0.01 | 1.58 | 2.38 | - |
| *Helgicirrha cari* |  | - | - | - | - | - | - | 0.28 |  | - | - | - | - | - | - | 0.63 |  | - | - | - | - | - | - | - |
| *Holothuria forskali* |  | - | - | - | - | - | - | 0.14 |  | - | - | - | - | - | - | - |  | - | - | - | - | - | - | - |
| *Inachus dorsettensis* |  | - | <0.01 | - | <0.01 | - | 0.27 | 0.14 |  | - | - | - | - | - | - | - |  | - | - | - | - | - | - | 0.68 |
| *Jaxea nocturna* |  | - | 0.19 | - | 0.24 | - | 0.55 | 0.42 |  | - | - | - | - | - | - | - |  | - | 0.17 | - | 0.19 | - | 1.19 | 0.68 |
| *Leuckartiara octona* |  | - | 0.10 | - | 0.16 | - | 0.27 | 1.12 |  | - | - | - | - | - | - | - |  | - | - | - | - | - | - | - |
| *Liocarcinus depurator* |  | - | <0.01 | - | <0.01 | - | 0.27 | 0.14 |  | - | - | - | - | - | - | 0.63 |  | - | - | - | - | - | - | 0.68 |
| *Liocarcinus maculatus* |  | - | - | - | - | - | - | - |  | - | 0.39 | - | 0.29 | - | 1.41 | 0.63 |  | - | - | - | - | - | - | - |
| *Liocarcinus vernalis* |  | - | 0.10 | - | 0.16 | - | 0.27 | 0.14 |  | - | 3.10 | - | 3.94 | - | 1.41 | 0.63 |  | - | - | - | - | - | - | - |
| *Liocarcinus zariquieyi* |  | - | 0.10 | - | 0.16 | - | 0.27 | 0.14 |  | - | - | - | - | - | - | - |  | - | - | - | - | - | - | - |
| *Lizzia blondina* |  | - | - | - | - | - | - | 2.52 |  | - | - | - | - | - | - | 3.13 |  | - | - | - | - | - | - | 0.68 |
| *Lucicutia flavicornis* |  | - | 0.05 | - | <0.01 | - | 1.37 | 1.26 |  | - | - | - | - | - | - | - |  | - | - | - | - | - | - | 5.41 |
| *Luidia sarsi* |  | - | - | - | - | - | - | - |  | - | - | - | - | - | - | 0.63 |  | - | - | - | - | - | - | - |
| *Magelona* sp. |  | - | - | - | - | - | - | 0.84 |  | - | - | - | - | - | - | - |  | - | - | - | - | - | - | - |
| *Mecynocera clausi* |  | - | 0.44 | - | <0.01 | - | 1.10 | 1.12 |  | - | - | - | - | - | - | - |  | - | - | - | - | - | - | - |
| *Meganyctiphanes norvegica* |  | 1.17 | - | 2.52 | - | 1.39 | - | - |  | 6.86 | 6.56 | 13.59 | 17.57 | 4.35 | 1.41 | 0.63 |  | 19.89 | 1.33 | 46.55 | 0.04 | 5.79 | 4.76 | 3.38 |
| *Merluccius merluccius* |  | - | - | - | - | - | - | 0.14 |  | - | - | - | - | - | - | - |  | - | - | - | - | - | - | - |
| *Mesocalanus tenuicornis* |  | - | <0.01 | - | <0.01 | - | 0.27 | 0.28 |  | - | - | - | - | - | - | - |  | - | - | - | - | - | - | - |
| Metanauplius (Euphausiid) |  | 0.06 | 0.05 | <0.01 | <0.01 | 0.46 | 0.27 | - |  | - | - | - | - | - | - | - |  | 0.16 | - | <0.01 | - | 0.53 | - | - |
| *Microsetella norvegica* |  | 0.42 | 0.68 | 0.01 | 0.02 | 2.08 | 2.20 | 2.81 |  | - | - | - | - | - | - | 2.50 |  | 1.21 | - | 0.03 | - | 4.74 | - | - |
| *Microsetella rosea* |  | 3.55 | 4.03 | 0.09 | 0.11 | 5.54 | 1.92 | - |  | 0.67 | - | 0.02 | - | 1.24 | - | - |  | 1.61 | 0.17 | 0.04 | <0.01 | 4.74 | 1.19 | - |
| *Muggiaea atlantica* |  | - | 0.39 | - | 0.65 | - | 0.55 | 3.09 |  | - | 0.77 | - | 1.52 | - | 1.41 | 3.13 |  | - | - | - | - | - | - | 0.68 |
| *Mullus barbatus* |  | - | - | - | - | - | - | 0.14 |  | - | - | - | - | - | - | - |  | - | - | - | - | - | - | 0.68 |
| *Nannocalanus minor* |  | - | 2.04 | - | <0.01 | - | 3.85 | 2.52 |  | - | 1.16 | - | <0.01 | - | 2.82 | 1.88 |  | - | - | - | - | - | - | - |
| *Nanomia bijunga* |  | - | - | - | - | - | - | 0.84 |  | - | - | - | - | - | - | 1.25 |  | - | - | - | - | - | - | - |
| *Nematoscelis megalops* |  | - | 13.36 | - | 27.42 | - | 1.92 | 1.82 |  | - | 15.06 | - | 28.16 | - | 2.82 | 2.50 |  | - | 4.49 | - | 10.92 | - | 3.57 | 3.38 |
| Nemertea ph. (Tubulanidae fam.) |  | - | - | - | - | - | - | 0.56 |  | - | - | - | - | - | - | 1.88 |  |  |  |  |  |  |  |  |
| *Obelia dichotoma* |  | - | - | - | - | - | - | 2.66 |  | - | - | - | - | - | - | 2.50 |  | - | - | - | - | - | - | 1.35 |
| *Obelia geniculata* |  | - | - | - | - | - | - | 0.14 |  | - | - | - | - | - | - | - |  | - | - | - | - | - | - | - |
| *Oncaea mediterranea* |  | - | 0.10 | - | <0.01 | - | 0.27 | 0.14 |  | - | - | - | - | - | - | - |  | - | - | - | - | - | - | - |
| *Oncaea scottodicarloi* |  | - | - | - | - | - | - | - |  | - | - | - | - | - | - | - |  | - | 7.15 | - | 0.02 | - | 1.19 | 0.68 |
| *Oncaea* spp. |  | 8.31 | 5.97 | 0.02 | 0.02 | 6.00 | 0.82 | - |  | 5.18 | 4.63 | 0.01 | 0.02 | 8.07 | 1.41 |  |  | 17.39 | - | 0.06 | - | 7.89 | - | - |
| *Oncaea venusta* |  | - | 1.26 | - | <0.01 | - | 0.55 | 0.42 |  | - | - | - | - | - | - | - |  | - | 7.15 | - | 0.02 | - | 1.19 | 0.68 |
| *Owenia fusiformis* |  | - | - | - | - | - | - | 0.14 |  | - | - | - | - | - | - | - |  | - | - | - | - | - | - | - |
| *Pagurus forbesii* |  | - | 0.05 | - | 0.05 | - | 0.55 | 0.28 |  | - | - | - | - | - | - | - |  | - | - | - | - | - | - | - |
| *Pagurus prideaux* |  | - | - | - | - | - | - | - |  | - | 0.39 | - | 0.49 | - | 1.41 | 0.63 |  | - | - | - | - | - | - | - |
| *Pandalina brevirostris* |  | - | 0.53 | - | 0.67 | - | 0.82 | 0.70 |  | - | 2.70 | - | 4.06 | - | 2.82 | 1.25 |  | - | 1.33 | - | 1.54 | - | 1.19 | 1.35 |
| *Paracalanus parvus* |  | - | 4.91 | - | 0.21 | - | 5.22 | 5.47 |  | - | 0.77 | - | <0.01 | - | 7.04 | 6.88 |  | - | 0.17 | - | <0.01 | - | 1.19 | 3.38 |
| *Paracentrotus lividus* |  | - | - | - | - | - | - | 0.98 |  | - | - | - | - | - | - | - |  | - | - | - | - | - | - | 0.68 |
| *Paraeuchaeta* sp. |  | - | <0.01 | - | <0.01 | - | 0.55 | 0.28 |  | 0.33 | 0.77 | 0.06 | 0.17 | 1.24 | 2.82 | - |  | 0.16 | 0.33 | 0.03 | 0.07 | 1.05 | 2.38 | - |
| *Pasiphaea sivado* |  | - | - | - | - | - | - | - |  | - | - | - | - | - | - | 0.63 |  | - | - | - | - | - | - | - |
| *Penilia avirostris* |  | 0.06 | - | <0.01 | - | 0.23 | - | - |  | - | - | - | - | - | - | - |  | - | - | - | - | - | - | - |
| *Philocheras bispinosus* |  | - | 3.89 | - | 4.22 | - | 1.65 | 1.68 |  | - | 17.37 | - | 23.75 | - | 5.63 | 3.75 |  | - | - | - | - | - | - | 0.68 |
| *Phoronis muelleri* |  | - | - | - | - | - | - | 0.28 |  | - | - | - | - | - | - | - |  | - | - | - | - | - | - | - |
| *Phrosina semilunata* |  | - | 0.24 | - | 0.55 | - | 0.27 | 0.14 |  | - | - | - | - | - | - | 1.25 |  | - | 1.16 | - | 2.83 | - | 1.19 | 0.68 |
| *Phyllodoce rosea* |  | - | - | - | - | - | - | 0.14 |  | - | - | - | - | - | - | - |  | - | - | - | - | - | - | - |
| *Pleuromamma borealis* |  | 0.76 | 2.09 | 0.04 | 0.11 | 1.85 | 4.12 | 2.24 |  | 4.52 | 3.09 | 0.24 | 0.21 | 4.97 | 4.26 | 1.25 |  | - | 2.33 | - | 0.07 | - | 1.19 | 3.38 |
| *Pleuromamma gracilis* |  | - | 0.19 | - | <0.01 | - | 1.10 | 0.84 |  | - | - | - | - | - | - | 0.63 |  | - | - | - | - | - | - | 3.38 |
| *Podocoryna areolata* |  | - | - | - | - | - | - | 1.12 |  | - | - | - | - | - | - | 1.25 |  | - | - | - | - | - | - | - |
| *Podon intermedius* |  | - | - | - | - | - | - | 0.70 |  | - | - | - | - | - | - | 1.25 |  | - | - | - | - | - | - | 1.35 |
| *Podon* sp. |  | - | - | - | - | - | - | - |  | 0.17 | - | <0.01 | - | 0.62 | - | - |  | - | - | - | - | - | - | - |
| Porifera ph. |  | - | - | - | - | - | - | 2.52 |  | - | - | - | - | - | - | 3.13 |  | - | - | - | - | - | - | 1.35 |
| *Porroecia spinirostris* |  | - | - | - | - | - | - | 0.14 |  | - | - | - | - | - | - | - |  | - | - | - | - | - | - | 0.68 |
| *Processa modica* |  | - | 0.10 | - | 0.11 | - | 0.82 | 0.56 |  | - | 0.39 | - | 0.49 | - | 1.41 | 1.25 |  | - | - | - | - | - | - | - |
| *Processa nouveli* |  | - | 7.48 | - | 8.76 | - | 3.85 | 2.24 |  | - | 1.16 | - | 1.75 | - | 1.41 | 2.50 |  | - | 1.33 | - | 3.13 | - | 1.19 | 2.03 |
| *Pseudosagitta lyra* |  | - | - | - | - | - | - | 0.42 |  | - | - | - | - | - | - | 0.63 |  | - | - | - | - | - | - | - |
| *Rhincalanus nasutus* |  | - | 0.73 | - | 0.03 | - | 1.10 | 0.84 |  | - | 4.63 | - | 0.18 | - | 2.82 | 1.25 |  | - | 0.83 | - | 0.02 | - | 2.38 | 2.03 |
| Siphonophorae ord. |  | 0.39 | - | 0.62 | - | 0.69 | - | - |  | 0.33 | - | 0.49 | - | 0.62 | - | - |  | - | - | - | - | - | - | - |
| *Scolecithricella dentata* |  | - | 0.68 | - | 0.13 | - | 0.55 | 0.28 |  | - | - | - | - | - | - | - |  | - | 0.33 | - | 0.07 | - | 1.19 | 0.68 |
| *Solenocera membranacea* |  | - | <0.01 | - | <0.01 | - | 0.55 | 0.56 |  | - | 0.39 | - | 0.29 | - | 1.41 | 1.25 |  | - | - | - | - | - | - | - |
| *Sphaeronectes koellikeri* |  | - | - | - | - | - | - | 1.40 |  | - | - | - | - | - | - | - |  | - | - | - | - | - | - | 2.70 |
| *Sphaeronectes irregularis* |  | - | - | - | - | - | - | 1.96 |  | - | - | - | - | - | - | - |  | - | - | - | - | - | - | - |
| *Spicara maena* |  | - | 0.15 | - | 0.07 | - | 0.82 | 1.68 |  | - | - | - | - | - | - | 1.25 |  | - | - | - | - | - | - | - |
| *Spiochaetopterus costarum* |  | - | - | - | - | - | - | 0.28 |  | - | - | - | - | - | - | - |  | - | - | - | - | - | - | - |
| *Spisula subtruncata* |  | - | 0.10 | - | <0.01 | - | 0.27 | 0.28 |  | - | - | - | - | - | - | - |  | - | - | - | - | - | - | - |
| *Subeucalanus pileatus* |  | - | 0.19 | - | <0.01 | - | 0.82 | 0.42 |  | - | - | - | - | - | - | - |  | - | - | - | - | - | - | - |
| *Temora* spp. |  | 0.22 | 0.39 | <0.01 | 0.02 | 1.62 | 1.92 | - |  | 0.17 | 0.39 | <0.01 | 0.02 | 0.62 | 1.41 | - |  | 0.32 | - | 0.02 | - | 1.58 | - | - |
| *Temora stylifera* |  | 0.45 | 0.73 | 0.02 | 0.03 | 2.08 | 2.47 | 0.56 |  | - | - | - | - | - | - | - |  | - | - | - | - | - | - | - |
| *Triconia dentipes* |  | - | 0.05 | - | <0.01 | - | 0.27 | 0.28 |  | - | 0.39 | - | <0.01 | - | 1.41 | 0.63 |  | - | 7.15 | - | 0.02 | - | 1.19 | 0.68 |
| *Trisopterus capelanus* |  | - | - | - | - | - | - | 0.28 |  | - | - | - | - | - | - | - |  | - | - | - | - | - | - | 0.68 |
| Unidentified amphipod |  | 0.06 | - | 0.19 | - | 0.46 | - | - |  | 0.17 | - | 0.53 | - | 0.62 | - | - |  | - | - | - | - | - | - | - |
| Unidentified calanoid |  | 6.55 | - | 0.27 | - | 5.54 | - | - |  | 7.86 | - | 0.30 | - | 9.32 | - | - |  | 7.97 | - | 0.35 | - | 6.32 | - | - |
| Unidentified decapod |  | 0.90 | 0.15 | 1.42 | 0.25 | 2.54 | 0.27 | - |  | 3.01 | - | 4.43 | - | 2.48 | - | - |  | 1.45 | - | 2.52 | - | 0.53 | - | - |
| Unidentified euphausiid |  | 15.67 | - | 33.55 | - | 2.31 | - | - |  | 23.75 | - | 47.08 | - | 9.94 | - | - |  | 4.19 | - | 9.80 | - | 3.16 | - | - |
| Unidentified malacostraca |  | 2.74 | - | 5.87 | - | 5.77 | - | - |  | 1.84 | - | 3.65 | - | 3.11 | - | - |  | 6.60 | - | 15.45 | - | 6.84 | - | - |
| Unidentified mysid |  | 0.03 | 0.05 | 0.06 | 0.11 | 0.23 | 0.27 | - |  | - | - | - | - | - | - | - |  | - | - | - | - | - | - | - |
| *Vibilia armata* |  | - | 0.05 | - | 0.11 | - | 0.27 | 0.14 |  | - | - | - | - | - | - | - |  | - | - | - | - | - | - | - |

**Table S5.** Diatom (Bacillariophyta ph.) families and species determined in stomach contents of sardinella (*S. aurita*), anchovy (*E. encrasicolus*) and sardine (*S. pilchardus*) with DNA metabarcoding. *N_S.aur_*, *N_E.enc_* and *N_S.pil_* denote the number of sardinella, anchovy and sardine stomach samples (and the percentage of samples, %) where the corresponding species (or diatom group) was found, respectively. %O is the frequency of occurrence. ‘und.sp’ means undetermined species within the indicated taxonomic group. ‘-’ means no item (0 or 0%). ‘Harmful’ column indicates the references that classified the corresponding taxa as toxic or potentially harmful algae for fish; and the ‘Rarity’ indicates the classification depending on the degree of distribution in the Mediterranean Sea according to Percopo, Siano, Cerino, Sarno, & Zingone (2011), defined as: A, abundant (>10^5^ cells l^-1^); F, frequent (10^4^–10^5^ cells l^-1^); R, rare (<10^4^ cells l^-1^); VR, very rare (<5x10^3^ cells l^-1^); ER, extremely rare, only observed in electron microscopy or serial dilution culture.

| ***Family*** | ***Prey species/group***  ***(DNA metabarcoding)*** |  | ***S. aurita*** | | |  | ***E. encrasicolus*** | | |  | ***S. pilchardus*** | | |  | ***Harmful*** | ***Rarity*** |
| --- | --- | --- | --- | --- | --- | --- | --- | --- | --- | --- | --- | --- | --- | --- | --- | --- |
|  |  |  | **N*_S.aur_* (%)** | | **%O** |  | **N*_E.enc_ (%)*** | | **%O** |  | **N*_S.pil_ (%)*** | | **%O** |  |  |  |
| **Bacillariaceae** | Bacillariaceae fam. (und.sp.) |  | 27 | (87.10) | 6.00 |  | 8 | (36.36) | 11.00 |  | 7 | (53.85) | 14.80 |  |  |  |
|  | *Cylindrotheca Closterium* |  | 17 | (54.84) | 1.89 |  | - | - | - |  | 1 | (7.69) | 0.80 |  | *(Hallegraeff, 2003) | F |
|  | *Nitzschia* sp. |  | 20 | (64.52) | 2.22 |  | 4 | (18.18) | 1.83 |  | 3 | (23.08) | 2.40 |  | *(Vila & Masó, 2005) | VR |
|  | *Psammodictyon constrictum* |  | 10 | (32.26) | 1.11 |  | - | - | - |  | - | - | - |  |  |  |
|  | *Pseudo-nitzschia americana* |  | 17 | (54.84) | 1.89 |  | 4 | (18.18) | 1.83 |  | - | - | - |  | *(Vila & Masó, 2005) |  |
|  | *Pseudo-nitzschia delicatissima* |  | 30 | (96.77) | 6.66 |  | 14 | (63.64) | 13.42 |  | 5 | (38.46) | 4.00 |  | *(Vila & Masó, 2005) | F |
|  | *Pseudo-nitzschia galaxiae* |  | 26 | (83.87) | 2.86 |  | 11 | (50.00) | 5.05 |  | 5 | (38.46) | 4.00 |  | *(Vila & Masó, 2005) | F |
|  | *Pseudo-nitzschia multiseries* |  | 21 | (67.74) | 2.33 |  | - | - | - |  | 1 | (7.69) | 0.80 |  | *(Vila & Masó, 2005) |  |
|  | *Pseudo-nitzschia multistriata* |  | 18 | (58.06) | 2.00 |  | 1 | (4.55) | 0.46 |  | - | - | - |  | *(Vila & Masó, 2005) | VR |
|  | *Pseudo-nitzschia* sp. |  | 24 | (77.42) | 2.66 |  | 7 | (31.82) | 3.21 |  | 2 | (15.38) | 1.60 |  | *(Vila & Masó, 2005) |  |
| **Catenulaceae** | *Amphora helenensis* |  | 17 | (54.84) | 1.89 |  | - | - | - |  | 2 | (15.38) | 1.60 |  |  |  |
| **Chaetocerotaceae** | Chaetocerotaceae fam. (und.sp.) |  | 23 | (74.19) | 2.55 |  | 1 | (4.55) | 0.46 |  | 1 | (7.69) | 0.80 |  |  |  |
|  | *Chaetoceros danicus* |  | 23 | (74.19) | 2.55 |  | 1 | (4.55) | 0.46 |  | 3 | (23.08) | 2.40 |  | *(Smayda, 2006) | VR |
|  | *Chaetoceros didymus* |  | 18 | (58.06) | 2.00 |  | - | - | - |  | - | - | - |  |  |  |
|  | *Chaetoceros socialis* |  | 24 | (77.42) | 2.66 |  | 6 | (27.27) | 2.75 |  | 6 | (46.15) | 4.80 |  | *(Hallegraeff, 2003) |  |
| **Corethraceae** | *Corethron hystrix* |  | 6 | (19.35) | 0.67 |  | - | - | - |  | 1 | (7.69) | 0.80 |  |  | R |
| **Coscinodiscaceae** | *Coscinodiscus radiatus* |  | 2 | (6.45) | 0.22 |  | - | - | - |  | - | - | - |  |  |  |
|  | *Coscinodiscus wailesii* |  | 2 | (6.45) | 0.22 |  | 1 | (4.55) | 0.46 |  | - | - | - |  | *(Lassus, Chomerat, Hess, & Nezan, 2016) | VR |
| **Cymatosiraceae** | *Arcocellulus mammifer* |  | 23 | (74.19) | 2.55 |  | 2 | (9.09) | 0.92 |  | 4 | (30.77) | 3.20 |  |  |  |
|  | *Papiliocellulus simplex* |  | 24 | (77.42) | 2.66 |  | 6 | (27.27) | 2.75 |  | 3 | (23.08) | 2.40 |  |  | ER |
| **Entomoneidaceae** | Entomoneidaceae fam. (und.sp.) |  | 15 | (48.39) | 1.66 |  | - | - | - |  | 2 | (15.38) | 1.60 |  |  |  |
| **Fragilariaceae** | *Asterionellopsis guyunusae* |  | - | - | - |  | 1 | (4.55) | 0.46 |  | - | - | - |  |  |  |
| **Grammatophoraceae** | Grammatophoraceae fam. (und.sp.) |  | 13 | (41.94) | 1.44 |  | - | - | - |  | - | - | - |  |  |  |
| **Hemiaulaceae** | *Cerataulina pelagica* |  | 21 | (67.74) | 2.33 |  | 6 | (27.27) | 2.75 |  | 4 | (30.77) | 3.20 |  | *(Hallegraeff, 2003) | F |
|  | *Eucampia cornuta* |  | 14 | (45.16) | 1.55 |  | 2 | (9.09) | 0.92 |  | - | - | - |  |  | VR |
|  | *Hemiaulus sinensis* |  | 11 | (35.48) | 1.22 |  | 1 | (4.55) | 0.46 |  | - | - | - |  |  |  |
| **Lauderiaceae** | *Lauderia annulate* |  | 20 | (64.52) | 2.22 |  | - | - | - |  | 2 | (15.38) | 1.60 |  |  |  |
| **Leptocylindraceae** | *Leptocylindrus danicus* |  | 23 | (74.19) | 2.55 |  | 12 | (54.55) | 5.50 |  | 3 | (23.08) | 2.40 |  |  | A |
| **Licmophoraceae** | *Licmophora abbreviata* |  | 8 | (25.81) | 0.89 |  | - | - | - |  | - | - | - |  |  |  |
| **Melosiraceae** | *Melosira varians* |  | 1 | (3.23) | 0.11 |  | 4 | (18.18) | 1.83 |  | - | - | - |  |  |  |
| **Naviculaceae** | Naviculaceae.fam (und.sp.) |  | 1 | (3.23) | 0.11 |  | - | - | - |  | 1 | (7.69) | 0.80 |  |  | ER |
| **Paraliaceae** | *Paralia sulcata* |  | 1 | (3.23) | 0.11 |  | 1 | (4.55) | 0.46 |  | - | - | - |  |  |  |
| **Pleurosigmataceae** | Pleurosigmataceae fam. (und.sp.) |  | 17 | (54.84) | 1.89 |  | 4 | (18.18) | 1.83 |  | 1 | (7.69) | 0.80 |  |  | VR |
| **Rhizosoleniaceae** | Rhizosoleniaceae fam. (und.sp.) |  | 21 | (67.74) | 2.33 |  | 2 | (9.09) | 0.92 |  | 4 | (30.77) | 3.20 |  |  |  |
|  | *Guinardia striata* |  | 23 | (74.19) | 2.55 |  | 3 | (13.64) | 1.38 |  | 1 | (7.69) | 0.80 |  |  | F |
|  | *Pseudosolenia calcar-avis* |  | 15 | (48.39) | 1.66 |  | 1 | (4.55) | 0.46 |  | - | - | - |  |  |  |
|  | *Rhizosolenia fallax* |  | 27 | (87.10) | 4.78 |  | 11 | (50.00) | 5.05 |  | 5 | (38.46) | 4.00 |  |  |  |
|  | *Rhizosolenia shrubsolei* |  | 25 | (80.65) | 2.77 |  | 9 | (40.91) | 4.13 |  | 7 | (53.85) | 7.60 |  |  |  |
| **Skeletonemataceae** | Skeletonemataceae fam. (und.sp.) |  | 24 | (77.42) | 2.66 |  | 10 | (45.45) | 4.59 |  | 4 | (30.77) | 3.20 |  |  |  |
|  | *Skeletonema potamos* |  | 13 | (41.94) | 1.44 |  | - | - | - |  | - | - | - |  |  |  |
| **Surirellaceae** | Surirellaceae fam. (und.sp.) |  | - | - | - |  | 1 | (4.55) | 0.46 |  | - | - | - |  |  |  |
| **Thalassionemataceae** | *Thalassionema frauenfeldii* |  | 4 | (12.91) | 0.44 |  | - | - | - |  | - | - | - |  |  | F |
| **Thalassiosiraceae.fam** | Thalassiosiraceae fam. (und.sp.) |  | 26 | (83.87) | 2.89 |  | 12 | (54.55) | 5.50 |  | 7 | (53.85) | 6.60 |  |  |  |
|  | *Conticribra weissflogii* |  | - | - | - |  | - | - | - |  | - | - | - |  |  |  |
|  | *Cyclotella atomus* |  | - | - | - |  | - | - | - |  | - | - | - |  |  |  |
|  | *Minidiscus trioculatus* |  | 30 | (96.77) | 3.33 |  | 19 | (86.36) | 11.35 |  | 12 | (92.31) | 14.20 |  |  | ER |
|  | *Planktoniella sol* |  | 18 | (58.06) | 2.00 |  | 3 | (13.64) | 1.38 |  | - | - | - |  |  |  |
|  | *Thalassiosira guillardi* |  | - | - | - |  | - | - | - |  | - | - | - |  |  |  |
|  | *Thalassiosira mediterranea* |  | 20 | (64.52) | 2.22 |  | 6 | (27.27) | 2.75 |  | 2 | (15.38) | 1.60 |  |  |  |
|  | *Thalassiosira oceanica* |  | 23 | (74.19) | 2.55 |  | 5 | (22.73) | 2.29 |  | 3 | (23.08) | 2.40 |  |  | ER |
|  | *Thalassiosira oestrupii* |  | 17 | (54.84) | 1.89 |  | - | - | - |  | 1 | (7.69) | 0.80 |  |  |  |
|  | *Thalassiosira pseudonana* |  | 1 | (3.23) | 0.11 |  | - | - | - |  | - | - | - |  |  |  |
| **Triceratiaceae.fam** | Triceratiaceae fam. (und.sp.) |  | 16 | (51.61) | 1.78 |  | 2 | (9.09) | 0.92 |  | - | - | - |  |  |  |
|  | *Odontella mobiliensis* |  | 8 | (25.81) | 0.89 |  | - | - | - |  | 1 | (7.69) | 0.80 |  |  |  |

**References**

Hallegraeff, G. (2003). Manual on harmful marine microalgae. In *Monogr. Oceanogr. Methodol* (Vol. 11). UNESCO Publishing.

Lassus, P., Chomerat, N., Hess, P., & Nezan, E. (2016). *Toxic and harmful microalgae of the World Ocean* (UNESCO. IO). International Society for the Study of Harmful Algae / Intergovernmental Oceanographic Commission of UNESCO.

Percopo, I., Siano, R., Cerino, F., Sarno, D., & Zingone, A. (2011). Phytoplankton diversity during the spring bloom in the northwestern Mediterranean Sea. *Botanica Marina*, *54*(3), 243–267. doi: 10.1515/bot.2011.033

Smayda, T. J. (2006). *Harmful algal bloom communities in Scottish coastal water: Relationship to fish farming and regional comparisons - A review*.

Vila, M., & Masó, M. (2005). Phytoplankton functional groups and harmful algal species in anthropogenically impacted waters of the NW Mediterranean Sea. *Scientia Marina*, *69*(1), 31–45.
